# Healthcare workers’ sustainable employability in relation to quality of care: an umbrella review

**DOI:** 10.1136/bmjopen-2024-095126

**Published:** 2025-09-08

**Authors:** Iris van de Voort, Ian Leistikow, Jan-Willem Weenink

**Affiliations:** 1 Erasmus School of Health Policy and Management, Erasmus University Rotterdam, Rotterdam, The Netherlands; 2 Health and Youth Care Inspectorate, Utrecht, The Netherlands

**Keywords:** Health Workforce, Quality in health care, Safety, Burnout, Fatigue, Job Satisfaction

## Abstract

**Abstract:**

**Objectives:**

There is a wealth of reviews investigating the relations between healthcare worker (HCW) variables and quality of care (QoC) outcomes. Individually, these reviews predominantly focus on one aspect relevant to HCWs’ functioning at work, unintentionally contributing to a scattered body of evidence. This umbrella review uses the concept of sustainable employability (SE)—a multidimensional construct that captures an individual’s long-term ability to function adequately at work and in the labour market—to integrate existing reviews on the topic, and to examine if and how HCWs’ SE is related to QoC.

**Design:**

An umbrella review of systematic reviews was conducted.

**Data sources:**

Systematically conducted reviews or meta-analyses of empirical primary studies (quantitative, qualitative or mixed methods) were included.

**Eligibility criteria for selecting studies:**

Reviews were eligible for inclusion if they included studies that focused on HCWs providing direct patient care; explored a relation between SE indicators and QoC outcomes; were peer-reviewed and published in an academic journal in either English or Dutch and were appraised as high-quality reviews.

**Data extraction and synthesis:**

We followed the Joanna Briggs Institute manual for Evidence synthesis when conducting this review. Nine verified indicators of SE, pertaining to health, well-being and competence domains of SE, were used to identify published reviews in Embase, Medline, PsycINFO and CINAHL up until 10 May 2024. Quality of reviews was critically appraised with the Joanna Briggs Institute Critical Appraisal Instrument. Data were extracted by one reviewer in a standardised form with a second reviewer verifying outcome data that directly informed our evidence statements.

**Results:**

55 high-quality reviews were included, and 6 SE indicators linked to 19 QoC outcome categories were identified, distinguishing a total of 50 unique relations—whether positive, negative, partial, absent or mixed. Although extensive, evidence is disproportionally represented, with reviews on ‘burn-out’ and ‘lack of knowledge/skills’ being over-represented and well-established. Only four reviews covered multiple SE domains simultaneously.

**Conclusions:**

When theoretically integrated, there is an impressive array of evidence showing the crucial role of HCWs’ SE for QoC. Researchers are advised to adopt more multidimensional perspectives and concepts to empirically validate the interrelatedness of individual HCW variables for QoC. Practitioners may use this overview to consider interventions that target multiple indicators of HCWs’ SE.

**STRENGTHS AND LIMITATIONS OF THIS STUDY**

- This umbrella review theoretically synthesises evidence from 55 other reviews, creating a holistic overview and understanding of the relations (ie, direction and extent) between healthcare worker variables and quality of care outcomes.
- Quality of reviews was assessed before inclusion, and only high-quality reviews were included in this synthesis.
- Inclusion criteria based on quality appraisal may have been too strict, as 61 articles had to be excluded in accordance with predefined quality criteria.
- Potentially relevant evidence from primary studies linking sustainable employability indicators to QoC outcomes that have not yet been systematically reviewed may have been missed but fell outside of our scope.

## Introduction

 Healthcare workers’ (HCWs) sustainable employability (SE)—described as an individual’s ability ‘to function as effectively, efficiently and healthily as possible within a given (un)employment context, now and in the future’^1^—is increasingly compromised.^2^,^4^ The current employment context for HCWs is characterised by intensified job demands as a result of increased demand for care due to different population health needs^5^ against a backdrop of personnel shortages due to high (work-related) absenteeism, turnover, expected mass-retirement and a decreased attractiveness of healthcare professions.^6^ Policy precautions are being made to counter the need for more HCWs (eg, telemedicine or AI), or to increase the (future) number of HCWs (eg, recruiting foreign-trained HCWs or delaying retirement ages).^7^,^9^ Despite efforts, it is still estimated that by 2030 there will be a net shortage of 10 million HCWs worldwide.^10^

This shortage translates into a vicious cycle of difficulties for the current healthcare workforce, increasingly challenging their long-term ability to function adequately at work (ie, SE).^3^ HCWs will be expected to function with higher workloads, frequent overtime, higher patient-to-clinician ratios, work–life interference, lack of breaks and inadequate recuperation times. Moreover, HCWs perceive their job resources, known to buffer job demands, to be low.^11^ As a result, many leave their jobs, or even the healthcare sector, dissatisfied and burned out, a trend exacerbated by the COVID-19 pandemic.^12^,^15^ Ensuing labour market shortages further increase difficulties for those who ‘remain’. On top of that, experienced HCWs are often replaced by new graduates, who often feel ill equipped, incompetent or unsupported to function adequately at work, which again contributes to the decision of many (about 17.5%) to leave their positions within a year.^16 17^

This negative spiral affects the delivery of safe, high-quality care.^18^ In the last decade or so, evidence and acknowledgement are accumulating how HCWs’ adequate functioning is imperative to healthcare quality. Scholars have stressed that ‘caring for the patient requires caring for the provider’^19^ and that ‘a fully staffed, psychologically well workforce is seen as ‘the’ foundational patient safety intervention’.^20^ Yet, the HCW variables that are being examined are often studied in isolation rather than in synergy. As such, the multidimensional and comprehensive character of HCWs’ long-term ability to function adequately is often deduced or simplified to a single variable, symptom or disease, such as burn-out. Consequently, interventions with a similar narrow focus may miss their potential to counteract persistent workforce challenges.

SE scholars have identified three domains (ie, health, well-being and employability) each hosting various indicators (eg, subjective health, job satisfaction, skill-gap) that distinctly affect individuals’ likelihood of remaining employed and individuals’ sustained ability to function adequately within employment.^21 22^ It follows that including multiple variables pertaining to different domains of HCWs’ SE is likely to sketch a more comprehensive picture of the relationship between HCWs’ SE and quality of care (QoC). However, despite scholarly work on the SE of HCWs,^23^,^26^ the relationship between HCWs’ SE and QoC has so far remained unexplored.

To that end, this study presents an overview of available evidence on the direct relationship between indicators of HCWs’ SE and QoC outcomes. Given the broad scope of this review and the current plethora of reviews in healthcare research, we conducted an umbrella review (ie, an overview of existing systematic reviews and meta-analyses). This umbrella review aims to create a solid evidence base that will help practitioners to understand the many ways in which HCWs’ SE—or lack thereof—may relate to QoC. We believe that a comprehensive understanding is a prerequisite for any endeavour that seeks to contribute to the SE of the healthcare workforce. Moreover, an overview of evidence provides researchers with concrete suggestions for future research to further supplement and refine the available evidence.

## Method

### Design

This umbrella review aims to present an overview of available evidence from multiple systematic reviews by answering the following research question: Is HCWs’ SE related to QoC? An umbrella review is appropriate when conducting research on a broad topic that has been the subject of multiple systematic reviews with potentially conflicting findings.^27^ In designing and conducting this umbrella review, we followed the Joanna Briggs Institute (JBI) manual for Evidence synthesis.^28^ The research team comprised researchers with backgrounds in occupational health, medicine and professional regulation, with the second and third authors having extensive experience with conducting literature reviews.

### Data sources and searches

An initial search in PubMed and PsycINFO was conducted on the term ‘sustainable employability’. No articles returned that focused on HCWs’ SE in relation to QoC. To overcome this limitation, the research team, in close consultation with a librarian holding a PhD in systematic review searches,^29^ created a search string based on the conceptualisation of SE by Fleuren *et al*,^21^ which includes nine verified indicators (ie, building blocks) of individuals’ SE: ‘perceived health status’, ‘work ability’, ‘fatigue’, ‘need for recovery’, ‘job satisfaction’, ‘motivation to work’, ‘perceived employability’, ‘skill-gap’ and ‘job performance’. The final search string included a combination of four key elements: indicators of SE (or their synonyms), QoC outcomes, HCWs, and reviews or meta-analyses. Subsequently, a systematic search of Embase, Medline, PsycINFO and CINAHL was performed up until 10 May 2024 to locate relevant (systematic) reviews and meta-analyses. The search was limited to English or Dutch reviews published in an academic journal. No restrictions on publication date were applied. In addition, we performed a backwards and forwards citation search. Online supplemental file 1 provides details on the used search strings for every database.

### Screening and selection

All titles and abstracts were initially screened for the inclusion/exclusion criteria (table 1) by the first author, with the third author screening a random selection of 10%. The full-text screening was similarly conducted with the first author screening full-text and the third author screening a random selection of 10%. Inter-rater agreement at both stages amounted to 96.6% and was considered satisfactory. Disagreements or doubtful cases were discussed with all authors until consensus was reached about inclusion. Screening was conducted in EndNote and took place between May and July 2024.

**Table 1:** Inclusion criteria

Categories	Description
Population	Healthcare workers providing direct patient care
Phenomena of interest/exposure	Identified and verified indicators (ie, building blocks) of SE (21) and their synonyms: ‘subjective health’, ‘need for recovery’, ‘fatigue’, ‘work ability’, ‘skill gap’, ‘performance’, ‘perceived employability’, ‘motivation’ and ‘job satisfaction’. Indicators should be perceived by healthcare workers, not others (eg, patients)
Outcomes	Quality of care outcomes
Context/setting	Any setting
Study type	Systematically conducted reviews or meta-analyses of empirical primary studies (quantitative, qualitative or mixed methods) that were of sufficient quality (ie, scoring at least 6/11 items in quality appraisal AND scoring a ‘yes’ in having conducted appropriate quality appraisal (item 5)
Publication type	English or Dutch peer-reviewed (systematic) reviews or meta-analyses published in academic journals and available in full text

### Quality appraisal

The JBI manual recommends only including reviews of high quality, and hence reviews considered eligible for inclusion were also subjected to critical appraisal. The JBI Critical Appraisal Instrument for Systematic Reviews and Research Synthesis^30^ was used to assess quality. This instrument comprises 11 questions pertaining to conducting methodologically sound systematic reviews, such as appropriate search and screening strategy (eg, two independent reviewers), appropriate quality appraisal, and methods to minimise errors in data extraction. Answer options for each item include ‘yes’, ‘no’, ‘unclear’ or ‘not applicable’. If a review scores ‘yes’ or ‘not applicable’ for a specific item, there is a positive score (ie, one point) on the checklist, with a maximum total score of 11. Despite the recommendation of the JBI appraisal instrument to only include high-quality reviews, the manual does not specify when a review is considered high quality. Instead, it instructs researchers to establish criteria beforehand. Accordingly, we predetermined that reviews scoring 5 or less points, or reviews that did not conduct quality appraisal (item 5), would be excluded from this umbrella review to avoid including findings of poor or unknown quality. If included (ie, scoring 6 or more and having conducted quality appraisal), reviews were considered high quality. Quality appraisal was conducted by the first author, with the third author appraising a random selection of 10%. Inter-rater agreement was 100% in terms of decisions to include or exclude. Differences on item-level were discussed and resolved.

### Data extraction and analysis

Data were extracted by the first author, with the third author verifying outcome data that directly informed our evidence statements.^31^,^33^ Data were extracted for study characteristics, including author/year, objective(s), review type and included designs, participants and context/setting, databases and date range, number of included studies, study designs, country of origin primary studies, quality appraisal methods including quality ratings, SE indicators and relevant QoC outcomes reported.

### Synthesis

Umbrella reviews do not aim to resynthesise the evidence, but rather provide an overview of existing syntheses on a specific topic. This review, in particular, aims to answer the question of how HCWs’ SE is related to QoC. As such, we have structured the extracted findings around indicators of SE that were identified through the review.

### Patient and public involvement

Patients or the public were not involved in the design, conduct, reporting or dissemination plans of our research.

## Results

**Figure 1:**
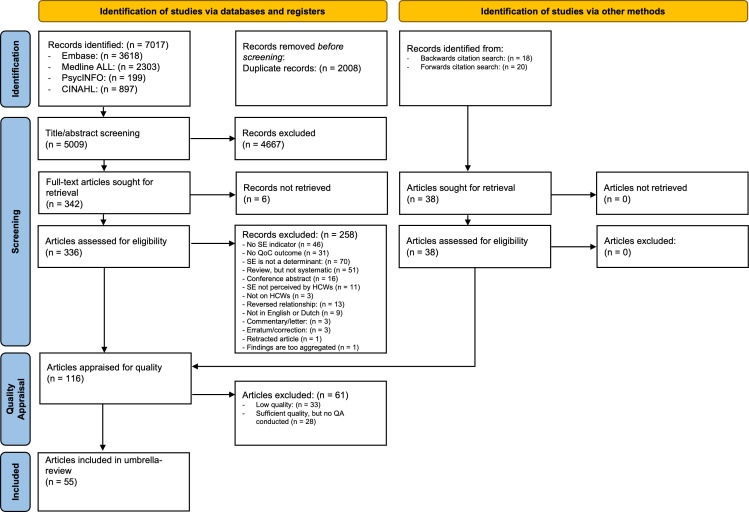
PRISMA flow chart. HCWs, healthcare workers; PRISMA, Preferred Reporting Items for Systematic Reviews and Meta-Analyses; QoC, quality of care; SE, sustainable employability.

The literature search yielded 7017 unique records after duplicates were removed, of which 78 articles met inclusion criteria after screening title/abstract and full text articles. In addition, the backwards and forwards citation search retrieved another 38 records. In total, 116 articles met the inclusion criteria and were subjected to quality appraisal (see figure 1 for a depiction of the process and exclusion reasons based on the Preferred Reporting Items for Systematic Reviews and Meta-Analyses flow chart).

### Quality of included reviews

Of 116 articles, 61 articles did not meet our predetermined quality criteria, meaning the final inclusion of this review amounts to 55 articles. Reviews that were excluded either scored negative for 6 or more items on the 11-item checklist—rendering the quality insufficient (33 studies) or scored positive on our checklist but did not appraise the quality of their own included studies (item 5) (28 studies)—rendering the quality of evidence as unknown. Most reviews lacked an appropriate search and screening strategy (69 studies, 59.4%)—usually due to not including a second independent reviewer—did not conduct quality appraisal with two or more reviewers (70 studies, 60.3%), or did not assess, consider or acknowledge potential publication bias (76 studies, 65.5%). Of the 55 reviews that passed quality appraisal criteria, 8 reviews (14.5%) scored positive on 6 or 7 items, 36 reviews (65.5%) scored positive on 8, 9 or 10 items and 11 reviews (20%) scored positive on all 11 items. Full details of the quality appraisal for all reviews can be found in online supplemental file 2.

### Characteristics of included reviews

Characteristics of the 55 included reviews are listed in online supplemental file 3. Included reviews were published between 2014 and 2024, with most reviews being published in 2016 (9; 16.4%) and 2021 (9; 16.4%). Reviews originated—as derived from the first author’s affiliation—from the USA (15), the UK (8), Australia (6), The Netherlands (3), Malaysia (3), Ireland (3), Italy (2), Slovenia (2), Canada (2), China (2), Ghana (1), Iran (1), Spain (1), Japan (1), Austria (1), Indonesia (1), Qatar (1), Norway (1) and Singapore (1). Based on the review typology of Sutton *et al*,^34^ included reviews belonged to the following ‘families’: 43 systematic reviews (80%), 6 qualitative reviews (10.9%), 4 traditional reviews (ie, narrative or integrative review) (7.3%), 1 mixed-methods review (1.8%) and 1 rapid review (1; 1.8%). Most reviews focused on healthcare professionals in general (27; 49.1%), others focused on nurses (15; 27.3%), physicians and/or residents (9; 16.4%), surgeons (3; 5.5%) and one focused on optometrists (1; 1.8%). Reviews either focused on specific indicators of SE (eg, burnout) that were examined in relation to several QoC outcomes (20; 36.4%), or reviews focused on specific QoC outcomes (eg, medication errors) and identified indicators of SE as one of the (many) contributing factors (35; 63.6%). There was a varied range in the number of primary studies included in each review (mean=31, median=21, range 5–170). Finally, reviews were published in 47 different journals, with 5 journals having published more than 1 review (ie, *International Journal of Nursing Studies* (5), *BMJ Open* (2), *Implementation Science* (2), *PLoS ONE* (2) and *Medical Care Research and Review* (2).

### Indicators of SE

With regard to SE, all domains (ie, health, well-being and employability) were represented in the included reviews, although not proportionately. Most articles concerned the employability domain of SE, with 34 reviews (61.8%) including either knowledge, skills, competence, ability or confidence to enact job-related tasks as determinants of, or factors affecting, QoC. In total, 20 reviews concerned the health domain of SE (36.4%) with 10 reviews indicating burnout, 8 reviews indicating fatigue and 2 reviews indicating mental ill health (eg, depression) as important determinants of, or factors affecting, QoC. Finally, 10 reviews concerned the well-being domain of SE (18.2%) with 6 reviews identifying work engagement and 4 reviews focusing on job satisfaction as determinants of, or factors affecting, QoC. While most reviews focused on indicators that pertain to only one domain (51; 92.7%), three reviews^35^,^37^ covered indicators of two separate domains, and one review covered indicators that pertain to all three domains of SE.^38^

### QoC outcomes

The included reviews covered a variety of 19 QoC outcome categories, such as patient outcomes (eg, mortality), patient satisfaction, HCWs perceived patient safety and safety events (eg, medical errors). Table 2 depicts all QoC outcomes. Some reviews that included a vast number of primary studies reported QoC in aggregated or unspecified form rather than how it was exactly measured (eg, errors). This is also indicated in table 2.

**Table 2:** Overview of evidence

	Indicators health domain SE		
Quality of care outcomes	Burnout	Mental ill-health	Fatigue
Safety events	Hall *et al*^39^; Salyers *et al*^40^; Tawfik *et al*^41^; de Jong *et al*^43^; Dewa *et al*^44^; Dewa *et al*^45^; Hodkinson *et al*^46^; Al-Ghunaim *et al*^47^; Al-Ghunaim *et al*^47^; Abraham *et al*^48^	Hall *et al*^39^; de Jong *et al*^43^; Pereira-Lima *et al*^49^	de Jong *et al*^43^; Cho *et al*^50^; Gates *et al*^53^
Medication errors	Tawfik *et al*^41^; Jun *et al*^42^	de Jong *et al* ^43^	Basil *et al*^35^; Parry *et al*^38^; Schroers *et al*^51^; Fathizadeh *et al*^52^
Suboptimal/missed care	Tawfik *et al*^41^; Tawfik *et al*^41^; Tawfik *et al*^41^; de Jong *et al*^43^; Dewa *et al*^44^; Abraham *et al*^48^		
Patient satisfaction/experience	Salyers *et al*^40^; Jun *et al*^42^; de Jong *et al*^43^; Dewa *et al*^45^; Hodkinson *et al*^46^; Al-Ghunaim *et al*^47^; Abraham *et al*^48^		
Patient enablement	Tawfik *et al* ^41^		
Patient treatment adherence			
Adherence to guidelines	Tawfik *et al*^41^; Jun *et al*^42^		
Reporting/speaking-up	Tawfik *et al*^41^; Jun *et al*^42^		Cho *et al* ^50^
Patient safety culture	Tawfik *et al* ^41^		
Malpractice claims	Tawfik *et al*^41^; Al-Ghunaim *et al*^47^		
Unfavourable patient outcomes	Tawfik *et al*^41^; Tawfik *et al*^41^; Jun *et al*^42^		Gates *et al*^53^; Reijmerink *et al*^54^
Access to care			
Patient safety activities			Cho *et al* ^50^
HCW perceived patient safety	Salyers *et al*^40^; Tawfik *et al*^41^; Jun *et al*^42^		C ho *et al* ^50^
HCW perceived quality of care	Salyers *et al*^40^; Tawfik *et al*^41^; Jun *et al*^42^; de Jong *et al*^43^; Dewa *et al*^45^; Al-Ghunaim *et al*^47^; Abraham *et al*^48^		Gates *et al* ^53^
Patient perceived quality of care	Jun *et al* ^42^		
Objectively measured quality of care			
Quality of care (unspecified)	T awfik *et al* ^41^		
Patient safety (unspecified)	Fekonja *et al* ^36^		Fekonja *et al* ^36^

	Indicators well-being domain SE		Indicators employability domain SE
Quality of care outcomes	Work engagement	Job satisfaction	Lack of knowledge/skills
Safety events	Janes *et al*^55^; Scheepers *et al*^58^	Scheepers *et al* ^58^	
Medication errors		Parry *et al* ^38^	Basil *et al*^35^; Parry *et al*^38^; Schroers *et al*^51^; Salmasi *et al*^74^; Thomas *et al*^75^
Suboptimal/missed care		Scheepers *et al*^58^; Scheepers *et al*^58^; Peng *et al*^59^	Carey *et al* ^71^
Patient satisfaction/ experience		Scheepers *et al* ^58^	O’Rorke *et al*^68^; Zhang *et al*^67^
Patient enablement			
Patient treatment adherence		Scheepers *et al* ^58^	
Adherence to guidelines			McFadden *et al*^69^; Baatiema *et al*^76^; Bromley *et al*^84^; Gallione *et al*^60^; Houghton *et al*^80^; Niño de Guzmán *et al*^81^; Slade *et al*^82^; Slade *et al*^82^; Smiddy *et al*^83^; Kelly *et al*^85^; Chatfield *et al*^86^; Cormican *et al*^77^; Craig *et al*^78^; Egerton *et al*^72^; Jun *et al*^61^; Ng *et al*^73^; Parajuli *et al*^79^; Toomey *et al*^70^
Reporting/speaking-up	Keyko *et al* ^57^	Okuyama *et al* ^37^	Okuyama *et al* ^37^ ; Peng *et al* ^59^ ; Balan *et al* ^87^ ; Hamed *et al* ^62^ ; Woo *et al* ^63^ ; De Angelis *et al* ^64^ ; Putri *et al* ^88^ ; Vaismoradi *et al* ^65^ ; Vrbnjak *et al* ^66^
Patient safety culture	Janes *et al* ^55^		
Malpractice claims			
Unfavourable patient outcomes	Wee *et al* ^56^	Scheepers *et al* ^58^	Zhang *et al* ^67^
Access to care			Pitzer *et al*^89^; Pitzer *et al*^89^
Patient safety activities			
HCW perceived patient safety			
HCW perceived quality of care	Keyko *et al*^57^; Wee *et al*^56^	Scheepers *et al* ^58^	
Patient perceived quality of care	Wee *et al* ^56^		
Objectively measured quality of care	Wee *et al* ^56^		
Quality of care (unspecified)	Wee *et al* ^56^		
Patient safety (unspecified)	Wee *et al* ^56^		Fekonja *et al* ^36^

Meaning of colours: **GREEN**:SE indicator increases the outcome; **RED**: SE indicator decreases the outcome; **PURPLE**: mixed evidence; **BLUE**: partial relation; **YELLOW**: no relation

HCW, healthcare worker; SE, sustainable employability.

### SE in relation to QoC

**Figure 2:**
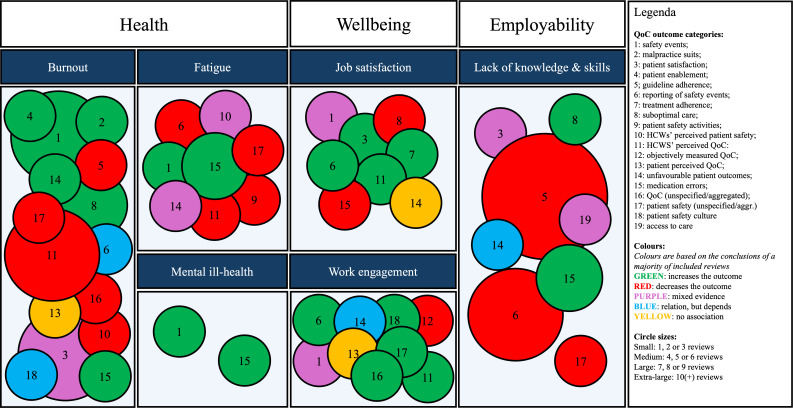
Extend and direction of evidence. HCWs, healthcare workers; QoC, quality of care.

From the included reviews, we identified 6 indicators of SE, 19 QoC outcomes and 50 unique SE-QoC combinations, as presented in table 2. In the remainder of this results section, we will discuss these combinations for every SE indicator separately. Figure 2 depicts the number of reviews that explored a specific SE-QoC combination and whether most reviews for every combination found a positive (green), negative (red), mixed (purple), partial (blue) or absent (yellow) relation. Mind that a positive and negative relation simply indicates that a specific indicator of SE, respectively, increases or decreases a specific QoC outcome, and not whether it constitutes a favourable/desirable outcome. However, at the end of every indicator paragraph, we do narratively summarise whether a specific SE indicator seems to positively (ie, favourably) or negatively (ie, unfavourably) affect QoC overall. Specific results extracted from reviews can be consulted in online supplemental file 4.

#### Burnout

Burnout was covered in 11 reviews. Reviews either focused on HCWs in general^39^,^41^ or focused on specific professions or settings, such as nurses,^36 42^ physicians,^43^,^46^ residents,^43 44^ surgeons^47^ or primary care providers.^48^ Reviews on burnout revealed relationships with 15 QoC outcomes.

**Safety events:** Nine reviews on burnout referred to safety events (ie, medical errors, near misses and adverse events). Four reviews associated burnout with a higher incidence of safety events.^39^,^4143 46^ One review found that burnout was associated with more patient safety incidents but only partially related to more errors, depending on the subdimension of the burnout scale used.^47^ Two reviews also established similar higher incidence of safety events, but only for self-perceived errors rather than errors based on chart audits.^44 45^ One review established that evidence was inconclusive.^48^

**Medication errors:** Two reviews on burnout and medication errors found that burnout resulted in more medication errors.^41 42^

**Suboptimal/missed care:** Four reviews on burnout referred to suboptimal care (eg, missed care or inappropriate care). Three reviews found that burnout, or at least one subdimension, resulted in a higher incidence of suboptimal care (practices).^43 44 48^ The review by Tawfik *et al*,^41^ with an extensive list of QoC outcomes, concluded that burnout—or at least one dimension of burnout—is associated with an increase in ordering inappropriate labs, inappropriate timing of discharge, suboptimal patient care practices, inappropriate use of patient restraints, lack of close monitoring, forgetting to convey information, not fully discussing treatment options, poor handoff quality and neglect of work. Emotional exhaustion and low feelings of personal accomplishment, two other subscales of the burnout measure, were associated with inappropriate antibiotic prescribing, while depersonalisation was related to fewer instances of inappropriate antibiotic prescribing. Similarly, burnout (aggregated) and low personal accomplishment (subscale of burnout measure) were associated with poor pain control, while emotional exhaustion was associated with better pain control. Finally, burnout was associated with fewer diagnostic delays.

**Patient satisfaction:** Seven reviews on burnout referred to patient satisfaction or experience. Three reviews concluded that burnout resulted in lower patient satisfaction.^40 42 46^ A fourth review partially corroborated these findings, with burnout being related to lower patient satisfaction for some subdimensions of the burnout scale.^45^ Two other reviews reported no association,^47 48^ and one review concluded that the evidence was mixed.^43^

**Patient enablement:** One review on burnout and patient enablement established that burnout was related to better patient enablement.^41^

**Adherence to guidelines:** Two reviews on burnout and adherence to guidelines found that burnout was related to poorer adherence.^41 42^

**Reporting/speaking-up:** Two reviews on burnout referred to the reporting of patient safety events or speaking-up behaviour. It was found that emotional exhaustion—a subscale of burnout—was associated with a lower frequency of events reported or fewer speaking-up behaviours, while there was no association found between low feelings of personal accomplishment—another subscale of burnout—and reporting of patient safety events.^42^ In addition, emotional exhaustion was related to fewer reports of near misses, whereas low feelings of personal accomplishment were related to more reports of near misses.^41^

**Patient safety culture:** One review on burnout and patient safety culture found that burnout and patient safety climate are partially associated, with overall burnout, low personal accomplishment and depersonalisation—subscales of burnout measure—being related to a lower patient safety score and emotional exhaustion—another subscale—being related to better safety climate scores.^41^

**Malpractice claims:** Two reviews on burnout and malpractice claims found that burnout was related to an increase in malpractice allegations or claims.^41 47^

**Unfavourable patient outcomes:** Two reviews on burnout referred to unfavourable patient outcomes. Burnout—or at least one subscale—was associated with increased patient mortality, increased morbidity, increased patient falls, increased healthcare-associated infections, increased urinary tract infections, increased surgical site infections and prolonged emergency department visits.^41 42^ Furthermore, the association between burnout and length of stay was negative (ie, shorter length of stay) when burnout was measured with one item, and the association was positive (ie, longer length of stay) when burnout was measured with the three subscales of the Maslach Burnout Inventory (MBI) measure.^41^ Similarly, emotional exhaustion and low feelings of personal accomplishment (ie, subscales of the MBI) were associated with increased posthospitalisation recovery time, while depersonalisation (ie, subscale of MBI) was associated with decreased posthospitalisation recovery time.^41^

**HCWs’ perceived patient safety:** Three reviews on HCWs perceived patient safety reported that burnout was associated with a decrease in provider-perceived patient safety.^40^,^42^

**HCWs’ perceived QoC:** Seven reviews on burnout referred to QoC as perceived by HCWs. Four reviews concluded that burnout is associated with lower provider-perceived QoC.^40^,^43^ Two other reviews partially confirmed this finding, although it was only for male rather than female surgeons^47^ or for some subscales of the burnout measure.^45^ A final review reported no association between burnout and provider-perceived QoC.^48^

**Patient perceived QoC:** One review concluded that there was no association between burnout and patient-perceived QoC.^42^

**QoC (aggregated/unspecified):** One review on burnout and QoC concluded that burnout results in lower QoC.^41^

**Patient safety (aggregated/unspecified):** One review on burnout and patient safety concluded that burnout results in lower patient safety.^36^

In summary, most evidence points to burnout having a negative impact on QoC.

#### Mental ill health

Mental ill health was discussed in three reviews and measured as ‘depressive symptoms’,^49^ ‘depression’^43^ and ‘mental health, distress, depression, anxiety and job stress’.^39^ Two reviews specifically focused on physicians,^43 49^ one on HCWs in general.^39^ Reviews on mental ill health covered two QoC outcomes.

**Safety events:** All three reviews on mental ill health referred to safety events. One meta-analysis of 11 studies and 21 517 physicians demonstrated that physicians with depressive symptoms have a significantly elevated risk of being involved in (perceived) medical errors.^49^ There was no significant difference between physicians and residents, but surgeons and US-based physicians had a higher risk of being involved in a medical error compared with physicians that were not based in the USA or working in other specialties. In addition, studies with a longitudinal design report a lower—but still elevated—risk of error involvement for physicians with depressive symptoms compared with studies with a cross-sectional design.^49^ Another systematic review reported that most primary studies included found a full association between mental ill health and increased errors.^39^ Finally, a third review concluded that errors were more likely to occur among depressed residents or to be reported by depressed residents in comparison to non-depressed residents.^43^

**Medication errors:** One review on mental ill health referred to medication errors. This review identified a significant relation between depressed physicians and the occurrence of medication errors.^43^

In summary, most evidence points to mental ill health having a negative impact on QoC.

#### Fatigue

Fatigue was covered in nine reviews, sometimes termed ‘sleep deprivation’, ‘sleepiness’ or ‘being tired or exhausted’. Although these terms have slightly different meanings, we will use the term fatigue hereafter. Five reviews focused on fatigue in nurses,^3638 50^,^52^ one on resident/physician fatigue,^43^ one on physician/surgeon fatigue,^53^ one specifically on surgeon fatigue^54^ and one on fatigue of HCWs working in the neonatal intensive care unit (NICU).^35^ Reviews found relationships for eight QoC outcomes.

**Safety events:** Three reviews on fatigue referred to safety events. Two reviews demonstrated that fatigue is significantly associated with more frequent self-perceived errors,^43 50^ while another review reported that evidence is mixed.^53^

**Medication errors**: Four reviews reported that HCWs perceived fatigue to be a cause or contributing factor to the occurrence of medication errors.^35 38 51 52^

**Reporting/speaking up:** One review found that fatigue is significantly associated with a lower frequency of reporting of safety events.^50^

**Unfavourable patient outcomes:** Two reviews on fatigue reported on unfavourable patient outcomes. One meta-analysis showed that there was no significant difference in mortality or postoperative complications between sleep-deprived and non-sleep-deprived surgeons.^53^ In terms of length of stay and intraoperative complications, most included primary studies did not report an association except for one study in cardiac surgery where sleep-deprived surgeons performed better than non-sleep deprived surgeons in terms of length of stay. In contrast, non-sleep deprived surgeons performed better in terms of intraoperative complications.^53^ Another review compared 110 real-life studies that explored the effect of fatigue on surgical outcomes and concluded that evidence is mixed, with 53 studies (48.2%) finding no effect, 39 studies (35.4%) finding worsened surgical outcomes when surgeons were fatigued, 9 studies (8.2%) finding an improvement when surgeons were fatigued and 9 studies (8.2%) finding mixed results.^54^

**Patient safety activities:** One review reported that nurse fatigue was significantly associated with nurses attending less to patient safety management activities.^50^

**HCW perceived patient safety:** One review reported that the evidence for the relationship between fatigue and nurse perceived patient safety is mixed, with one primary study reporting how it leads to lower perceived patient safety and another study reporting no association.^50^

**HCW perceived QoC**: One review found that physician fatigue is significantly related to decreased physician-perceived QoC.^53^

**Patient safety (aggregated/unspecified):** One review reported that nurses indicated fatigue—attributed to a 12-hour work-day—to be a contributor to worse patient safety during triage in an emergency department.^36^

In summary, most evidence points to fatigue having a negative impact on QoC.

#### Work engagement

Work engagement was covered in four reviews. Two reviews focused on work engagement of HCWs in general,^55 56^ one on work engagement of nurses^57^ and one specifically on physicians’ work engagement.^58^ Reviews on work engagement found relationships with nine QoC outcomes.

**Safety events:** Two reviews on work engagement reported on relationships with safety events. One review conducted a meta-analysis and found that work engagement was associated with fewer medical errors and adverse events.^55^ Another review found mixed evidence, with two primary studies confirming that work engagement leads to fewer medical errors, while two other primary studies reported no association.^58^

**Reporting/speaking up**: One review reported that work engagement significantly increased nurses’ voice behaviour.^57^

**Patient safety culture:** One review found that most of their included primary studies reported an improvement of patient safety culture when HCWs were engaged.^55^

**Unfavourable patient outcomes:** One review found that work engagement decreased 7-day patient mortality, although only for engaged nurses and not for engaged physicians.^56^

**HCW perceived QoC:** Two reviews on work engagement found that work engagement significantly increased QoC when self-assessed by HCWs.^56 57^

**Patient perceived QoC:** One review found that there is no longer an association between work engagement and QoC when the latter is assessed by patients.^56^

**Objectively measured QoC**: Similarly, one review found that when QoC is objectively measured, work engagement actually leads to lower QoC.^56^ Unfortunately, the authors do not state how QoC was exactly objectively measured.

**QoC (unspecified/aggregated):** One review reported how work engagement improves QoC.^56^

**Patient safety (unspecified/aggregated):** One review reported how work engagement enhances patient safety, although it was not specified how patient safety was measured.^56^

In summary, most evidence points to work engagement having a positive impact on QoC.

#### Job satisfaction

Four reviews identified job or career satisfaction as a relevant factor or determinant of QoC. Two reviews focused on nurses’ job satisfaction,^38 59^ one on physicians’ job satisfaction,^58^ and one on HCWs in general.^37^ Reviews on job satisfaction found relationships with eight QoC outcomes.

**Safety events:** One review reported how the association between job satisfaction and medical errors was mixed, with two primary studies finding that physicians’ job satisfaction is associated with fewer medical errors, while two other studies found no association.^58^

**Medication errors**: One review found that nurses who were less satisfied about their work perceived themselves to make more medication administration errors.^38^

**Suboptimal care:** Two reviews on job satisfaction referred to suboptimal care. One review reported that physicians reported less suboptimal care when they had higher levels of job satisfaction.^58^ The same review concluded that there is, however, no association established between physicians’ job satisfaction and physicians avoiding or offering superfluous care in consultations.^58^ Another review concluded that nurse career dissatisfaction resulted in more missed nursing care.^59^

**Patient satisfaction:** One review established that physicians’ job or career satisfaction is associated with higher patient satisfaction.^58^

**Treatment adherence:** One review found that physicians’ job or career satisfaction is associated with increased patient adherence to treatment.^58^

**Reporting/speaking-up:** One review concluded that HCWs who were more satisfied with their jobs exhibited more speaking-up behaviour.^37^

**Unfavourable patient outcomes:** One review concluded that there is no association between physicians’ job satisfaction and patients’ self-reported pain and depressive symptoms.^58^

**HCW perceived QoC:** One review reported that physicians with higher job satisfaction also reported better self-perceived QoC.^58^

In summary, most evidence points to job satisfaction having a positive impact on QoC.

#### Lack of knowledge and skills

Knowledge and skills, or a lack thereof, were identified as a relevant factor or determinant of QoC by 32 reviews. Some reviews focused on the knowledge and skills of specific professions, such as nurses,^3651 52 60^,^66^ physicians,^46 67^ surgeons,^68^ birth attendants,^69^ optometrists,^70^ primary care clinicians^3871^,^73^ or focused on specific settings, such as the NICU,^35^ South-East Asia^74^ or long-term care.^65^ Reviews on knowledge and/or skills found relationships with eight QoC outcomes.

**Medication errors:** Five reviews on knowledge and skills referred to medication errors. Four reviews identified a lack of knowledge and/or skills as a perceived cause or contributing factor to medication (administration) errors.^35 51 74 75^ Examples of knowledge deficits referred to drug interactions, safe dilutions and proper dosages. One review^38^ found mixed evidence, with two primary studies reporting that nurses with greater knowledge or expertise were less likely to violate medication protocols or to make non-severe errors, whereas two other primary studies within that review reported that greater knowledge among nurses actually contributed to more medication errors.

**Suboptimal care:** One review on knowledge and/or skills identified (a lack of) knowledge and/or skills of HCWs as a barrier and/or enabler to the delivery of optimal or high-quality palliative care.^71^

**Patient satisfaction:** Two reviews on knowledge and skills referred to patient satisfaction. One review found that surgeons’ communication and interpersonal skills positively contributed to patient satisfaction.^68^ However, another review reported that physicians’ empathy as a communicative competence is associated with increased patient satisfaction if empathy is measured by a researcher counting empathic verbal responses and not by self-perception.^67^

**Adherence to guidelines**: 17 reviews on knowledge and skills found relationships with the adherence to evidence-based practice (EBP) or clinical practice guidelines (CPGs). 15 reviews identified (a lack of) knowledge and/or skills as a barrier or facilitator to the adherence to EBP or CPGs pertaining to midwifery care,^69^ (acute) stroke care,^76^,^78^ palliative care,^79^ eye care,^70^ respiratory infectious diseases,^80^ breast cancer,^81^ low back pain (LBP),^82^ musculoskeletal pain,^73^ osteoarthritis,^72^ hand hygiene,^83^ colonoscopic cancer screening in African Americans^84^ or EBP, standards and CPGs in general.^60 61 85^ Examples of knowledge or skills deficits included a lack of clinical skills as required in CPG, a lack of knowledge on the recommended treatments in CPGs, or simply a lack of knowledge on the existence of CPGs. In addition, one review reported a more nuanced finding for the management of low back pain, as it appeared that general practitioners, and not so much allied health clinicians (eg, physiotherapists), lacked confidence in their ability to assess LBP as recommended in CPGs.^82^ Another review relayed that HCWs do not believe that knowledge, or a lack thereof, is a contributing factor in low hand hygiene compliance rates.^86^

**Reporting/speaking-up:** Nine reviews on knowledge and skills found relationships with HCWs’ reporting of safety events or speaking-up behaviour. All nine reviews concluded that (a lack of) knowledge and skills is a barrier or facilitator for the reporting of adverse drug reactions (ADRs),^64 87 88^ incidents,^62^ missed nursing care,^59^ errors,^63 65^ medication errors^66^ and speaking up for patient safety.^37^ Examples of knowledge deficits inhibiting reporting or speaking-up behaviour pertained to clinical knowledge, insufficient knowledge on what constitutes missed care, or not knowing how to report an ADR.

**Unfavourable patient outcomes:** One review found that physicians’ display of empathy was associated with patients’ functional status (here: less anxiety), again only if measured by a researcher counting empathic verbal responses rather than physicians’ self-perception.^67^

**Access to care:** One review found that a lack of knowledge of HCWs about palliative care was a barrier to patients’ access to palliative care.^89^ However, patients’ access to palliative care was lower when non-palliative care clinicians reported high self-confidence in palliative care competence.^89^

**Patient safety (unspecified/aggregated):** One review established that (a lack of) knowledge and/or skills of HCWs was a barrier and/or enabler to overall patient safety during nurse triage in the ER.^36^

In summary, most evidence points to a lack of knowledge or skills having a negative impact on QoC.

## Discussion

### Key findings

This umbrella review underlines an overall positive effect of HCWs’ SE on QoC. From 55 reviews, we have identified and gathered evidence for a direct relation between 6 indicators of SE and 19 QoC outcomes, displaying a total of 50 unique combinations between the two, whether positive, negative, partial, absent or mixed. This relatively young body of evidence (2014–2024) mostly covered (a lack of) knowledge and/or skills as important for QoC outcomes, followed by burnout. Relatively few reviews focused on other mental ill health conditions, such as depression or anxiety, and none were identified for any physical form of (occupational) disability. The principal focus on the development and retention of knowledge and skills may be unsurprising, as this component of HCWs’ SE is inherently challenged by new insights and knowledge developments in healthcare. Unsurprisingly then, most of our included reviews that identified knowledge and skills as important to QoC focused on its relationship with (new) guideline adoption and adherence. In contrast, we did not identify reviews that assessed to what extent a lack of knowledge or skills played a role in safety events, such as adverse events or medical errors, signalling an important gap in systematically gathered literature. Burnout has gained incredible academic traction in the last two decades, amounting to over 6000 book and article publications as of 2009.^90^ Despite the well-deserved attention, the preoccupation with burnout in healthcare may devalue or distract from other manifestations of HCW impairment or distress, such as compassion fatigue, secondary traumatic stress disorder, moral injury or cognitive dissonance, none of which were mentioned in reviews included here.^91^ Furthermore, although individuals’ ability to function adequately at work throughout careers (ie, SE) is argued to be multidimensional (ie, health, well-being, employability) with different dimensions interacting with one another, it is striking that only four included reviews considered indicators belonging to two or three SE domains. Also, although the quality of reviews included in this umbrella review was adequate, it was striking that we had to exclude 61 reviews due to important methodological weaknesses.

Overall, we report that for most QoC outcomes, burnout, mental ill health, fatigue and a lack of knowledge and/or skills of HCWs are negatively related to QoC, while HCWs’ job satisfaction and work engagement are positively related to QoC. However, there were a few findings that require further nuance and contextualisation. First, we found mixed evidence for the relationship between burnout and patient satisfaction. It seems that burnout leads to lower patient satisfaction when it concerns professions that engage frequently in day-to-day or long-term direct patient care, such as nurses or physicians in dialysis care. In contrast, there is no association between burnout and patient satisfaction when it concerns surgeons or primary care physicians, perhaps because their long-term interactions with patients are fewer. Interestingly, however, is that there is no association reported between nurse burnout and patient perceived QoC, suggesting that patients make inferences about their caretakers that influence their satisfaction or perception of QoC differently. The latter may also explain why work engagement of HCWs does not affect patient perceived QoC. Second, a few inconclusive findings between SE indicators and QoC are largely attributable to a lack of adequate and consistent measurement of either indicator or outcome. For example, the link between surgeon fatigue and patient outcomes remains inconclusive, probably as there are many variant operationalisations of fatigue that are often measured with proxies, such as time of operation, which up until now remain unvalidated. Similarly, the inconsistent effect of job satisfaction on safety events, such as medical errors, is most likely due to countless variations in type of error (eg, diagnostic, medication, surgical) and source (self-report or audits) measured.

### Limitations

We experienced some difficulty extracting findings from some reviews. Many reviews, especially those that include a lot of primary studies, aggregate specific findings into concepts (eg, merely ‘QoC’) to make it easier for the reader to grasp the abundance of information. However, as categorisations are not always universal and specific findings from primary studies are not always reported, it makes it harder for an umbrella review to extract exact findings. To overcome this limitation, we specifically reported when QoC outcome measures concerned aggregate rather than concrete findings. Moreover, our decision to exclude low-quality reviews or reviews that did not conduct quality appraisal themselves may be regarded as too strict given the high number of exclusions at that stage. However, as 55 reviews still amounted to a significant number of reviews included, we believed it was best not to mix results from well-conducted reviews with results from reviews with questionable or unknown quality. On top of that, it is highly likely that there is significant overlap between reviews in terms of included primary studies, increasing our confidence that we have not excluded too many relevant primary studies. Finally, we might have missed relevant evidence from primary studies linking SE indicators to QoC outcomes that have not yet been systematically reviewed.

### Future research

As measurement instruments exist to measure SE,^92 93^ we advise scholars to familiarise themselves with SE and to empirically examine the concept in relation to QoC outcomes, ideally in longitudinal form to capture how SE develops over time and to understand the direction of causality. In contrast to separate indicators of SE, examining SE as a multidimensional construct has the potential to show how different indicators or subcomponents of SE may interplay to affect HCWs’ SE, and as such provides for more nuanced and integrative results. Moreover, as is evident from table 2, systematic reviews may fill the existing gaps between HCWs’ variables and QoC outcomes that continue to exist, particularly for indicators that have received no or relatively little attention, such as musculoskeletal disorders, depression, moral injury, secondary trauma disorder and work ability. In a similar vein, qualitative reviews were relatively scarce and did not always explore how or why SE indicators related to QoC outcomes. This is primarily due to SE indicators being but one out of many factors identified in these reviews to affect certain QoC outcomes. Future research is encouraged to also explore how or why SE exactly relates to QoC.

### Implications for practice

Healthcare organisations often struggle in balancing their institutional interests to continue the delivery of care with their responsibility to maintain a well-rested and motivated workforce.^20^ In practice, with ongoing patient demands, it is likely and perhaps understandable that ‘the (ongoing) needs of the system often over-ride staff well-being at work’,^94^ as was apparent during the COVID-19 pandemic. This is not likely to change soon or easily, due to a persistent ‘serve or sacrifice’ culture in healthcare, that is equally reinforced by society.^95^ This constant balancing act requires a rebalance, as advocated elsewhere, where society, policy-makers, regulators and HCWs should reflect on ‘the current risk to patient safety posed by a depleted and unwell workforce versus the likely gains of prioritising the needs of the workforce going forward’.^20^ The concrete and substantiated findings of this umbrella review may aid that reflection, because it shows the many ways in which health workforce metrics affect QoC outcomes, and in turn, also how QoC may be improved through intervening on concrete SE indicators. A first essential step to be prioritised by healthcare organisations is to routinely measure and monitor HCW outcomes through human resource metrics. Ideally, metrics that may inform all three domains of HCWs’ SE, such as health screenings or measurements,^96 97^ well-being metrics,^20^ or continuous professional development systems.^98^ Mind that these metrics should not just be used for measurement, but primarily serve as an accelerator for dialogue or reflection for HCWs and their managers to improve and sustain HCWs’ employability. Second, we recommend healthcare organisations to facilitate close collaboration between their quality and human resource departments to enable relevant data to be shared and intervened on. In other words, we recommend human resource metrics to be regarded as QoC indicators.^99^ Third, regulators in healthcare are advised to monitor not just whether such metrics are collected, but especially whether these are used for organisational learning and development purposes (eg, new policies or tailored interventions) to further avoid a ‘tick-box exercise’.^20^ Finally, all these steps require an engaged workforce, and hence it is recommended to involve HCWs to understand and capture their local needs in particular contexts or for specific professions.^100^ These actions may seem labourious, but our evidence suggests that a sustainably happy, healthy and well-equipped workforce has the potential to sustain safe and quality care for both present and future patients.

## Supplementary material

10.1136/bmjopen-2024-095126 online supplemental file 1

10.1136/bmjopen-2024-095126 online supplemental file 2

10.1136/bmjopen-2024-095126 online supplemental file 3

10.1136/bmjopen-2024-095126 online supplemental file 4

## Data Availability

Data are available on reasonable request.
