# Supplementary material for: Healthcare workers’ sustainable employability in relation to quality of care: an umbrella review
Source: BMJ Open. 2025 Sep 8;15(9):e095126. doi: 10.1136/bmjopen-2024-095126 (PMC12421189; doi:10.1136/bmjopen-2024-095126)
Supplement: online supplemental file 1 [file bmjopen-15-9-s001.pdf]

Supplemental file 1: Search string - sustainable employability and quality of care
Latest update: May 10, 2024

**Database searched**	**via**	**Years of coverage**	**Records**	**Records after duplicates removed**
Embase	Embase.com	1971 - Present	3618	3589
Medline ALL	Ovid	1946 - Present	2303	938
PsycINFO	Web of Knowledge	1975 - Present	199	93
CINAHL	Wiley	1992 - Present	897	389
**Total**			**7017**	**5009**

*Science Citation Index Expanded (1975-present) ; Social Sciences Citation Index (1975-present) ; Arts & Humanities Citation Index (1975-present) ; Conference Proceedings Citation Index- Science (1990-present) ; Conference Proceedings Citation Index- Social Science & Humanities (1990-present) ; Emerging Sources Citation Index (2015-present)

embase.com

('health care quality'/mj/de OR 'patient safety'/mj/exp OR 'medical error'/mj/exp OR 'protocol compliance'/mj OR 'patient satisfaction'/mj OR 'quality indicator'/mj OR 'quality indicators'/mj OR 'total quality management'/mj OR falling/mj OR complication/exp/mj OR 'adverse event'/exp/mj OR 'professional-patient relationship'/exp/mj OR (((care OR healthcare) NEAR/6 (qualit* OR safety OR performance* OR productivit*)) OR (patient* NEAR/3 (safet* OR harm OR risk OR injur*)) OR ((safety OR quality) NEAR/3 (indicator* OR metric* OR Measurement* OR indicator* OR culture* OR perception* OR perceive* OR incident* OR self-report* OR improve*)) OR malpractice* OR ((medical* OR diagnos* OR medicat* OR surg*) NEAR/3 (error* OR mistake*)) OR ((patient* OR accident*) NEAR/3 (fall*)) OR ((protocol* OR guideline*) NEAR/3 (complian* OR adherence*)) OR ((patient* OR client* OR resident*) NEAR/3 (satisf* OR dissatisf* OR complaint* OR experience* OR improve* OR wellbeing* OR well-being* OR outcome* OR measure* OR centered* OR centred* OR relation*)) OR complication* OR adverse* OR near-miss* OR noncomplian* OR nonadher* OR non-complian* OR non-adher* OR exacerbat*):ti) **AND** ('career mobility'/exp OR employability/de OR burnout/de OR 'professional burnout'/de OR 'job stress'/exp OR 'job satisfaction'/de OR 'psychological resilience'/de OR 'subjective well being'/de OR 'subjective wellbeing'/de OR presenteeism/de OR (perception/de AND ('job performance'/de)) OR ((Motivation/de OR 'mental stress'/de OR stress/de OR morality/de) **AND** ('health personnel attitude'/exp OR 'health care personnel'/exp)) OR (((career* OR workforce* OR working-life* OR work) NEAR/3 (mobilit*

OR ladder OR advance* OR progression* OR opportunit* OR trajector* OR movement* OR satisf* OR sustain*)) OR clinical-ladder* OR employabilit* OR ((external* OR internal*) NEAR/3 mobility) OR burnout* OR burn-out OR ((job OR occupation* OR work*) NEAR/3 (stress* OR fatigue OR insecur* OR perception* OR satisf*)) OR occupational-health OR resilien* OR (compassion NEAR/3 fatigue) OR ((professional* OR nurse* OR doctor* OR physician* OR provider* OR clinician*) NEAR/6 (subjective OR emotional* OR perceive* OR perception* OR feeling) NEAR/6 (performance* OR respect OR recogn* OR appreciat* OR knowledge* OR skill* OR competen*)) OR Skill-gap* OR ((professional* OR nurse* OR nursing OR doctor* OR physician* OR provider* OR occupation* OR employee* OR staff* OR surgeon*) NEAR/6 (wellbeing OR well-being OR ill-being OR wellness OR stress OR suicide OR anxiety OR depressi* OR mood OR sleep-deprivat* OR sleep-disrupt OR fatigue* OR sleepiness OR alertness OR exhaustion OR sleep-deficien* OR sleep-debt* OR sleepless* OR energy OR vigilan* OR fit-to-perform* OR Motivati* OR morale* OR engagement OR challenged OR thriving OR vigor OR dedication OR satisf* OR health-status* OR general-health* OR perceived-health OR subjective-health OR vitality OR fitness OR Work*-abilit* OR work*-function* OR function*-capacit* OR capacity-to-work* OR disruptive-behav* OR incivil* OR commit* OR respected* OR recogni* OR appreciat* OR leader* OR joy OR happiness OR happy)) OR presenteeism* OR secondary-victim-syndrome* OR Need-for-recover*):Ab,ti) AND (review/exp OR 'meta analysis'/de OR ((systematic* NEAR/3 review*) OR meta-analy*):ab,ti OR review:ti)

Medline ALL Ovid

(*Quality of Health Care/ OR * Patient Safety/ OR * Medical Errors/ OR * Guideline Adherence/ OR * Patient Satisfaction/ OR * Quality Indicators, Health Care/ OR * Total Quality Management/ OR *Accidental Falls/ OR exp *Professional-Patient Relations/ OR (((care OR healthcare) ADJ6 (qualit* OR safety OR performance* OR productivit*)) OR (patient* ADJ3 (safet* OR harm OR risk OR injur*)) OR ((safety OR quality) ADJ3 (indicator* OR metric* OR Measurement* OR indicator* OR culture* OR perception* OR perceive* OR incident* OR self-report* OR improve*)) OR malpractice* OR ((medical* OR diagnos* OR medicat* OR surg*) ADJ3 (error* OR mistake*)) OR ((patient* OR accident*) ADJ3 (fall*)) OR ((protocol* OR guideline*) ADJ3 (complian* OR adherence*)) OR ((patient* OR client* OR resident*) ADJ3 (satisf* OR dissatisf* OR complaint* OR experience* OR improve* OR wellbeing* OR well-being* OR outcome* OR measure* OR centered* OR centred* OR relation*)) OR complication* OR adverse* OR near-miss* OR noncomplian* OR nonadher* OR non-complian* OR non-adher* OR exacerbat*).ti.) AND (Career Mobility/ OR Burnout, Professional/ OR Burnout, Psychological/ OR Occupational Stress/ OR Job Satisfaction/ OR Resilience, Psychological/ OR Presenteeism/ OR ((Motivation/ OR Stress, Psychological/ OR Morals/) AND (Attitude of Health Personnel/ OR exp Health Personnel/)) OR (((career* OR workforce* OR working-life* OR work) ADJ3 (mobilit* OR ladder OR advance* OR progression* OR opportunit* OR trajector* OR movement* OR satisf* OR sustain*)) OR clinical-ladder* OR employabilit* OR ((external* OR internal*) ADJ3 mobility) OR burnout* OR burn-out OR ((job OR occupation* OR work*) ADJ3 (stress* OR fatigue OR insecur* OR perception* OR satisf*)) OR occupational-health OR resilien* OR (compassion ADJ3 fatigue)

OR ((professional* OR nurse* OR doctor* OR physician* OR provider* OR clinician*) ADJ6 (subjective OR emotional* OR perceive* OR perception* OR feeling) ADJ6 (performance* OR respect OR recogn* OR appreciat* OR knowledge* OR skill* OR competen*)) OR Skill-gap* OR ((professional* OR nurse* OR nursing OR doctor* OR physician* OR provider* OR occupation* OR employee* OR staff* OR surgeon*) ADJ6 (wellbeing OR well-being OR ill-being OR wellness OR stress OR suicide OR anxiety OR depressi* OR mood OR sleep-deprivat* OR sleep-disrupt OR fatigue* OR sleepiness OR alertness OR exhaustion OR sleep-deficien* OR sleep-debt* OR sleepless* OR energy OR vigilan* OR fit-to-perform* OR Motivati* OR morale* OR engagement OR challenged OR thriving OR vigor OR dedication OR satisf* OR health-status* OR general-health* OR perceived-health OR subjective-health OR vitality OR fitness OR Work*-abilit* OR work*-function* OR function*-capacit* OR capacity-to-work* OR disruptive-behav* OR incivil* OR commit* OR respected* OR recogni* OR appreciat* OR leader* OR joy OR happiness OR happy)) OR presenteeism* OR secondary-victim-syndrome* OR Need-for-recover*).ab,ti.) AND (systematic review/ OR review/ OR Meta-Analysis/ OR ((systematic* ADJ3 review*) OR meta-analy*).ab,ti. OR review:ti)

Cinahl

(MM Quality of Health Care OR MM Patient Safety OR MM Medical Errors OR MM Guideline Adherence OR MM Patient Satisfaction+ OR MM Quality Indicators, Health Care+ OR MM Total Quality Management+ OR MM Accidental Falls+ OR MM Professional-Patient Relations+ OR TI(((care OR healthcare) N5 (qualit* OR safety OR performance* OR productivit*)) OR (patient* N2 (safet* OR harm OR risk OR injur*)) OR ((safety OR quality) N2 (indicator* OR metric* OR Measurement* OR indicator* OR culture* OR perception* OR perceive* OR incident* OR self-report* OR improve*)) OR malpractice* OR ((medical* OR diagnos* OR medicat* OR surg*) N2 (error* OR mistake*)) OR ((patient* OR accident*) N2 (fall*)) OR ((protocol* OR guideline*) N2 (complian* OR adherence*)) OR ((patient* OR client* OR resident*) N2 (satisf* OR dissatisf* OR complaint* OR experience* OR improve* OR wellbeing* OR well-being* OR outcome* OR measure* OR centered* OR centred* OR relation*)) OR complication* OR adverse* OR near-miss* OR noncomplian* OR nonadher* OR non-complian* OR non-adher* OR exacerbat*)) AND (MH Career Mobility+ OR MH Burnout, Professional+ OR MH Burnout, Psychological+ OR MH Occupational Stress+ OR MH Job Satisfaction+ OR MH Resilience, Psychological+ OR MH Presenteeism+ OR ((MH Motivation+ OR MH Stress, Psychological+ OR MH Morals+) AND (MH Attitude of Health Personnel+ OR MH Health Personnel+)) OR TI(((career* OR workforce* OR working-life* OR work) N2 (mobilit* OR ladder OR advance* OR progression* OR opportunit* OR trajector* OR movement* OR satisf* OR sustain*)) OR clinical-ladder* OR employabilit* OR ((external* OR internal*) N2 mobility) OR burnout* OR burn-out OR ((job OR occupation* OR work*) N2 (stress* OR fatigue OR insecur* OR perception* OR satisf*)) OR occupational-health OR resilien* OR (compassion N2 fatigue) OR ((professional* OR nurse* OR doctor* OR physician* OR provider* OR clinician*) N5 (subjective OR emotional* OR perceive* OR perception* OR feeling) N5 (performance* OR respect OR recogn* OR appreciat* OR knowledge* OR skill* OR competen*)) OR Skill-gap* OR ((professional* OR nurse* OR nursing OR doctor* OR physician* OR provider* OR occupation* OR employee* OR staff* OR surgeon*) N5

(wellbeing OR well-being OR ill-being OR wellness OR stress OR suicide OR anxiety OR depressi* OR mood OR sleep-deprivat* OR sleep-disrupt OR fatigue* OR sleepiness OR alertness OR exhaustion OR sleep-deficien* OR sleep-debt* OR sleepless* OR energy OR vigilan* OR fit-to-perform* OR Motivati* OR morale* OR engagement OR challenged OR thriving OR vigor OR dedication OR satisf* OR health-status* OR general-health* OR perceived-health OR subjective-health OR vitality OR fitness OR Work*-abilit* OR work*-function* OR function*-capacit* OR capacity-to-work* OR disruptive-behav* OR incivil* OR commit* OR respected* OR recogni* OR appreciat* OR leader* OR joy OR happiness OR happy)) OR presenteeism* OR secondary-victim-syndrome* OR Need-for-recover*) OR AB(((career* OR workforce* OR working-life* OR work) N2 (mobilit* OR ladder OR advance* OR progression* OR opportunit* OR trajector* OR movement* OR satisf* OR sustain*)) OR clinical-ladder* OR employabilit* OR ((external* OR internal*) N2 mobility) OR burnout* OR burn-out OR ((job OR occupation* OR work*) N2 (stress* OR fatigue OR insecur* OR perception* OR satisf*)) OR occupational-health OR resilien* OR (compassion N2 fatigue) OR ((professional* OR nurse* OR doctor* OR physician* OR provider* OR clinician*) N5 (subjective OR emotional* OR perceive* OR perception* OR feeling) N5 (performance* OR respect OR recogn* OR appreciat* OR knowledge* OR skill* OR competen*)) OR Skill-gap* OR ((professional* OR nurse* OR nursing OR doctor* OR physician* OR provider* OR occupation* OR employee* OR staff* OR surgeon*) N5 (wellbeing OR well-being OR ill-being OR wellness OR stress OR suicide OR anxiety OR depressi* OR mood OR sleep-deprivat* OR sleep-disrupt OR fatigue* OR sleepiness OR alertness OR exhaustion OR sleep-deficien* OR sleep-debt* OR sleepless* OR energy OR vigilan* OR fit-to-perform* OR Motivati* OR morale* OR engagement OR challenged OR thriving OR vigor OR dedication OR satisf* OR health-status* OR general-health* OR perceived-health OR subjective-health OR vitality OR fitness OR Work*-abilit* OR work*-function* OR function*-capacit* OR capacity-to-work* OR disruptive-behav* OR incivil* OR commit* OR respected* OR recogni* OR appreciat* OR leader* OR joy OR happiness OR happy)) OR presenteeism* OR secondary-victim-syndrome* OR Need-for-recover*)) AND (MM systematic review+ OR MM review+ OR MM Meta-Analysis+ OR TI((systematic* N2 review*) OR meta-analy*) OR AB((systematic* N2 review*) OR meta-analy*) OR TI(review))

PsycInfo

(*Quality of Care/ OR * Patient Safety/ OR * Client Satisfaction/ OR (((care OR healthcare) ADJ6 (qualit* OR safety OR performance* OR productivit*)) OR (patient* ADJ3 (safet* OR harm OR risk OR injur*)) OR ((safety OR quality) ADJ3 (indicator* OR metric* OR Measurement* OR indicator* OR culture* OR perception* OR perceive* OR incident* OR self-report* OR improve*)) OR malpractice* OR ((medical* OR diagnos* OR medicat* OR surg*) ADJ3 (error* OR mistake*)) OR ((patient* OR accident*) ADJ3 (fall*)) OR ((protocol* OR guideline*) ADJ3 (complian* OR adherence*)) OR ((patient* OR client* OR resident*) ADJ3 (satisf* OR dissatisf* OR complaint* OR experience* OR improve* OR wellbeing* OR well-being* OR outcome* OR measure* OR centered* OR centred* OR relation*)) OR complication* OR adverse* OR near-miss* OR noncomplian* OR nonadher* OR non-complian* OR non-adher* OR exacerbat*).ti.) AND (Occupational Mobility/ OR Occupational

Stress/ OR Job Satisfaction/ OR Resilience, Psychological/ OR ((Motivation/ OR Psychological Stress/ OR Morality/) AND (Health Personnel Attitudes/ OR exp Health Personnel/)) OR (((career* OR workforce* OR working-life* OR work) ADJ3 (mobilit* OR ladder OR advance* OR progression* OR opportunit* OR trajector* OR movement* OR satisf* OR sustain*)) OR clinical-ladder* OR employabilit* OR ((external* OR internal*) ADJ3 mobility) OR burnout* OR burn-out OR ((job OR occupation* OR work*) ADJ3 (stress* OR fatigue OR insecur* OR perception* OR satisf*)) OR occupational-health OR resilien* OR (compassion ADJ3 fatigue) OR ((professional* OR nurse* OR doctor* OR physician* OR provider* OR clinician*) ADJ6 (subjective OR emotional* OR perceive* OR perception* OR feeling) ADJ6 (performance* OR respect OR recogn* OR appreciat* OR knowledge* OR skill* OR competen*)) OR Skill-gap* OR ((professional* OR nurse* OR nursing OR doctor* OR physician* OR provider* OR occupation* OR employee* OR staff* OR surgeon*) ADJ6 (wellbeing OR well-being OR ill-being OR wellness OR stress OR suicide OR anxiety OR depressi* OR mood OR sleep-deprivat* OR sleep-disrupt OR fatigue* OR sleepiness OR alertness OR exhaustion OR sleep-deficien* OR sleep-debt* OR sleepless* OR energy OR vigilan* OR fit-to-perform* OR Motivati* OR morale* OR engagement OR challenged OR thriving OR vigor OR dedication OR satisf* OR health-status* OR general-health* OR perceived-health OR subjective-health OR vitality OR fitness OR Work*-abilit* OR work*-function* OR function*-capacit* OR capacity-to-work* OR disruptive-behav* OR incivil* OR commit* OR respected* OR recogni* OR appreciat* OR leader* OR joy OR happiness OR happy)) OR presenteeism* OR secondary-victim-syndrome* OR Need-for-recover*).ab,ti.) AND (systematic review/ OR review/ OR Meta Analysis/ OR ((systematic* ADJ3 review*) OR meta-analy*).ab,ti. OR review:ti)
