# Supplementary material for: Healthcare workers’ sustainable employability in relation to quality of care: an umbrella review
Source: BMJ Open. 2025 Sep 8;15(9):e095126. doi: 10.1136/bmjopen-2024-095126 (PMC12421189; doi:10.1136/bmjopen-2024-095126)
Supplement: online supplemental file 2 [file bmjopen-15-9-s002.pdf]

**Supplemental file 2:** Quality appraisal

Exclusion if:

- Scoring less than 6 items
- Quality appraisal is not conducted (properly)

Y=Yes, N=No, U=Unclear, N/A=Not Applicable

- Yes and N/A = positive score (1)
- No or Unclear = negative score (0)

| | ***Items*** | | | | | | | | | | | |
|---|---|---|---|---|---|---|---|---|---|---|---|---|
| ***Authors*** | **1** | **2** | **3** | **4** | **5** | **6** | **7** | **8** | **9** | **10** | **11** | **Score** |
| *Abraham et al. (2020)* | Y | Y | U | Y | Y | Y | Y | N/A | Y | Y | Y | 10 |
| *Abubakar et al. (2014)* | N | Y | N | Y | N | N | N | N/A | N | N | Y | 4 |
| *Al-Ghunaim et al. (2022)* | Y | Y | Y | Y | Y | Y | Y | Y | Y | Y | Y | 11 |
| *Alansari et al. (2013)* | N | N | N | Y | N | U | N | N | N | N | Y | 2 |
| *Alomari et al. (2015)* | Y | Y | N | Y | N | N | U | Y | Y | N | N | 5 |
| *Alsulami et al. (2013)* | Y | Y | N | Y | N | U | N | Y | N | N | Y | 5 |
| *Baatiema et al. (2017)* | Y | Y | N | Y | Y | Y | Y | Y | N | Y | Y | 9 |
| *Balan (2021)* | Y | Y | Y | Y | Y | Y | Y | Y | N | Y | N | 9 |
| *Bamforth et al. (2023)* | Y | Y | N | Y | N | N | Y | Y | Y | Y | Y | 8 |
| *Basil et al. (2022)* | Y | Y | Y | Y | Y | Y | Y | Y | Y | Y | Y | 11 |
| *Bell et al. (2022)* | Y | Y | U | Y | N | N | U | Y | N | Y | Y | 6 |
| *Birks & Watt (2007)* | Y | Y | N | N | N | N | U | N | N | Y | Y | 4 |
| *Brady et al. (2009)* | Y | Y | N | Y | N | U | N | N | N | N | Y | 4 |
| *Bromley et al. (2015)* | Y | Y | Y | Y | Y | Y | Y | Y | Y | Y | Y | 11 |
| *Brown et al. (2016)* | N | N | N | N | N | N | U | U | N | Y | N | 1 |
| *Carey et al. (2019)* | Y | Y | U | Y | Y | Y | Y | N/A | N | Y | Y | 9 |

| | | | | | | | | | | | | |
|---|---|---|---|---|---|---|---|---|---|---|---|---|
| *Chatfield et al. (2017)* | Y | Y | Y | Y | Y | Y | Y | N/A | N | Y | Y | 10 |
| *Cho & Steege (2021)* | Y | Y | Y | Y | Y | Y | Y | N/A | N | Y | Y | 10 |
| *Cordeiro et al. (2022)* | Y | Y | Y | Y | N | N | Y | Y | N | Y | Y | 9 |
| *Cormican et al. (2023)* | Y | Y | N | Y | Y | N | Y | Y | N | Y | Y | 8 |
| *Craig et al. (2016)* | Y | Y | N | Y | Y | Y | Y | Y | N | Y | N | 8 |
| *Cruz & Pincus (2002)* | Y | N | N | Y | N | N | N | U | N | Y | Y | 4 |
| *De Angelis et al. (2016)* | Y | Y | Y | Y | Y | Y | Y | Y | Y | Y | Y | 11 |
| *De Jong et al. (2016)* | Y | Y | Y | N | Y | Y | Y | Y | N | Y | Y | 9 |
| *Derksen et al. (2013)* | N | Y | N | Y | N | N | N | N | N | Y | Y | 4 |
| *Dewa et al. (2017a)* | Y | Y | Y | Y | Y | Y | N | N/A | Y | N | Y | 9 |
| *Dewa et al. (2017b)* | Y | Y | Y | Y | Y | U | N | N/A | N | Y | Y | 8 |
| *Egerton et al. (2017)* | Y | Y | Y | Y | Y | Y | Y | Y | N | Y | Y | 10 |
| *Erasmus et al. (2010)* | Y | Y | Y | Y | N | N | Y | Y | N | Y | N | 7 |
| *Fathizadeh et al. (2024)* | Y | N | Y | Y | Y | Y | Y | N/A | Y | Y | N | 8 |
| *Fekonja et al. (2023)* | Y | Y | N | Y | Y | Y | Y | Y | N | Y | Y | 9 |
| *Fien et al. (2021)* | Y | Y | Y | Y | N | N | Y | Y | Y | Y | Y | 9 |
| *Filby et al. (2016)* | Y | Y | Y | Y | N | N | N | N | Y | Y | N | 6 |
| *Frith (2013)* | Y | Y | N | Y | N | N | U | U | N | Y | N | 4 |
| *Gallione et al. (2022)* | Y | Y | U | Y | Y | Y | Y | Y | N | Y | N | 7 |
| *Garcia et al. (2019)* | Y | N | Y | Y | N | Y | Y | Y | Y | N | N | 7 |
| *Gates et al. (2018)* | Y | Y | Y | Y | Y | Y | Y | Y | Y | Y | Y | 11 |
| *Goodrich and Lazenby (2022)* | N | N | N | Y | N | U | Y | Y | N | N | Y | 4 |
| *Gustafsson & Eriksson* | Y | Y | N | Y | N | Y | N | Y | N | N | Y | 6 |
| *Hall et al. (2016)* | Y | Y | N | Y | Y | Y | Y | Y | Y | N | Y | 9 |
| *Hamed & Konstandinidis (2022)* | Y | Y | N | Y | Y | U | Y | N/A | N | N | Y | 7 |
| *Hewitt (2010)* | Y | Y | N | Y | N | N | N | U | N | Y | N | 4 |

| | | | | | | | | | | | | |
|---|---|---|---|---|---|---|---|---|---|---|---|---|
| *Hodkinson et al. (2022)* | Y | Y | Y | Y | Y | Y | Y | Y | Y | Y | Y | 11 |
| *Houghton et al. (2020)* | Y | Y | Y | N | Y | Y | Y | Y | N | Y | Y | 9 |
| *Ippolito et al. (2024)* | Y | N | N | N | N | N | N | U | N | Y | Y | 3 |
| *Janes et al. (2021)* | Y | Y | Y | Y | Y | Y | Y | Y | Y | N | Y | 10 |
| *Johnson et al. (2018)* | Y | N | N | Y | N | N | N | U | N | Y | Y | 4 |
| *Jun et al. (2016)* | Y | Y | U | Y | Y | U | U | N | Y | Y | Y | 7 |
| *Jun et al. (2021)* | Y | Y | U | Y | Y | Y | Y | N/A | Y | Y | Y | 10 |
| *Keers et al. (2013)* | Y | Y | U | Y | N | N | Y | Y | Y | N | Y | 7 |
| *Kelly et al. (2023)* | Y | Y | Y | Y | Y | Y | Y | Y | Y | Y | N | 10 |
| *Keyko et al. (2016)* | Y | Y | Y | Y | Y | N | N | Y | U | Y | Y | 8 |
| *Koda et al. (2021)* | Y | Y | N | N | N | N | U | Y | Y | Y | Y | 6 |
| *Kohanová et al. (2023)* | Y | Y | Y | Y | N | N | Y | Y | N | Y | Y | 8 |
| *Koleva (2020)* | Y | Y | N | Y | N | N | U | U | Y | Y | Y | 6 |
| *Li et al. (2023)* | Y | Y | N | Y | N | N | N | U | N | N | N | 3 |
| *Lineaweaver et al. (2018)* | Y | U | N | U | N | N | U | U | N | Y | N | 2 |
| *Liu et al. 2008* | Y | Y | N | Y | N | U | N | Y | N | N | Y | 5 |
| *Lopez-Gonzalez et al. (2009)* | Y | N | N | Y | N | N | N | Y | N | N | Y | 4 |
| *Mangory et al. (2021)* | Y | Y | N | Y | N | N | N | N/A | Y | N | Y | 6 |
| *Martin et al. (2023)* | Y | Y | Y | Y | N | N | Y | N | N | Y | Y | 7 |
| *McConville and Hooven (2021)* | Y | Y | N | Y | N | N | U | U | N | Y | Y | 5 |
| *McFadden et al. (2020)* | Y | Y | Y | Y | Y | N | N | Y | N | Y | Y | 8 |
| *McTaggart & Walker (2022)* | Y | Y | U | N | N | N | N | N/A | N | Y | N | 4 |
| *Mitchell (2002)* | Y | Y | N | Y | N | N | N | Y | N | N | Y | 5 |
| *Mossburg & Himmelfarb (2021)* | Y | Y | N | Y | N | N | Y | N/A | N | N | Y | 6 |
| *Moyo et al. (2023)* | Y | Y | Y | Y | N | Y | U | Y | N | N | Y | 8 |
| *Mulchandani & Kakkar (2018/2019)* | Y | N | N | N | N | N | N | U | N | Y | N | 2 |

| | | | | | | | | | | | | |
|---|---|---|---|---|---|---|---|---|---|---|---|---|
| *Ng et al. (2021)* | Y | Y | Y | Y | Y | Y | N | Y | N | Y | Y | 8 |
| *Nijkamp & Foran (2021)* | Y | Y | N | Y | N | U | N | N | Y | Y | Y | 6 |
| *Niño de Guzmán et al. (2020)* | Y | Y | N | Y | Y | Y | Y | Y | N | N | Y | 8 |
| *Nuairi et al. (2022)* | Y | Y | N | Y | N | N | N | N | N | N | N | 3 |
| *O'rorke et al. (2022)* | Y | Y | N | Y | Y | U | N | Y | N | Y | Y | 7 |
| *Okuyama et al. (2014)* | Y | Y | Y | Y | Y | U | Y | Y | N | Y | Y | 9 |
| *Owoc et al. (2022)* | Y | Y | U | Y | N | N | Y | Y | Y | Y | N | 7 |
| *Pappa & Dafogianni (2022)* | Y | N | N | N | N | N | N | N | N | N | Y | 2 |
| *Parajuli & Hupcey (2021)* | Y | Y | U | Y | Y | U | Y | U | N | Y | Y | 7 |
| *Parry et al. (2015)* | Y | Y | U | Y | Y | U | Y | Y | N | N | N | 6 |
| *Peng et al. (2023)* | Y | Y | Y | Y | Y | Y | N | N/A | N | N | Y | 8 |
| *Pereira-Lima et al. (2019)* | Y | Y | Y | Y | Y | Y | Y | Y | Y | Y | Y | 11 |
| *Pitzer et al. (2024)* | Y | Y | Y | Y | Y | Y | Y | Y | N | Y | Y | 10 |
| *Putri et al. (2024)* | Y | Y | Y | Y | Y | Y | Y | Y | N | Y | Y | 10 |
| *Rahmah et al. (2022)* | Y | Y | N | Y | N | N | N | N | N | N | N | 3 |
| *Rahmat & Karuppannan (2021)* | Y | Y | U | Y | N | N | N | N | N | Y | N | 4 |
| *Rathert et al. (2018)* | Y | Y | Y | N | N | N | U | N/A | N | Y | Y | 6 |
| *Reijmerink et al. (2024)* | Y | Y | N | Y | Y | N | Y | Y | N | Y | N | 7 |
| *Saintsing et al. (2011)* | Y | Y | N | Y | N | N | N | U | N | Y | N | 4 |
| *Salehi et al. (2021)* | Y | Y | Y | Y | N | U | Y | N/A | Y | Y | Y | 9 |
| *Salmasi et al. (2015)* | Y | Y | U | Y | Y | U | U | N/A | N | Y | N | 6 |
| *Salyers et al. (2017)* | Y | Y | U | Y | Y | Y | Y | Y | Y | N | Y | 9 |
| *Sattar et al. (2020)* | Y | Y | Y | Y | N | Y | Y | Y | Y | N | Y | 10 |
| *Scheepers et al. (2015)* | Y | Y | Y | Y | Y | Y | Y | N/A | Y | Y | Y | 11 |
| *Schroers et al. (2021)* | Y | Y | U | Y | Y | U | N | N/A | Y | Y | Y | 8 |
| *Scott et al. (2022)* | Y | Y | N | Y | N | N | N | Y | Y | Y | N | 6 |

| | | | | | | | | | | | | |
|---|---|---|---|---|---|---|---|---|---|---|---|---|
| *Shayan et al. (2018)* | Y | Y | N | Y | N | U | Y | N | N | Y | Y | 6 |
| *Siokal et al. (2023)* | Y | N | N | Y | N | N | N | N | N | N | Y | 3 |
| *Slade et al. (2016)* | Y | Y | Y | Y | Y | Y | Y | Y | N | Y | Y | 10 |
| *Smiddy et al. (2015)* | Y | Y | U | Y | Y | Y | Y | Y | Y | N | N | 8 |
| *Smolders et al. (2008)* | N | Y | Y | Y | N | N | Y | Y | Y | Y | N | 7 |
| *Stewart (1995)* | Y | Y | N | N | N | N | N | Y | Y | Y | Y | 6 |
| *Sturm et al. (2011)* | Y | Y | N | Y | N | U | Y | Y | N | Y | Y | 7 |
| *Tawfik et al. (2019)* | Y | Y | Y | Y | Y | N/A | Y | Y | Y | N | Y | 10 |
| *Tetteh et al. (2022)* | Y | Y | U | Y | N | N | N | N | N | Y | Y | 5 |
| *Thomas et al. (2019)* | Y | Y | Y | Y | Y | Y | Y | Y | N | Y | N | 9 |
| *Toomey et al. (2021)* | Y | Y | Y | Y | Y | Y | Y | Y | Y | Y | Y | 11 |
| *Tully et al. (2009)* | Y | Y | U | Y | N | U | Y | Y | N | Y | Y | 7 |
| *Vaismoradi et al. (2020a)* | Y | Y | Y | Y | Y | Y | Y | Y | Y | Y | Y | 11 |
| *Vaismoradi et al. (2020b)* | Y | N | Y | Y | N | Y | N | Y | N | N | Y | 6 |
| *Vally et al. (2023)* | Y | Y | N | Y | N | N | N | N | N | Y | Y | 5 |
| *Vrbnjak et al. (2016)* | Y | Y | N | Y | Y | Y | Y | Y | N | Y | Y | 9 |
| *Wee & Lai (2022)* | Y | Y | Y | Y | Y | Y | N | Y | Y | Y | Y | 10 |
| *Whelehan et al. (2020)* | Y | Y | U | Y | N | N | N | N | N | Y | Y | 5 |
| *Williams & Skinner (2003)* | Y | Y | N | Y | N | N | N | N | N | Y | Y | 5 |
| *Wong et al. (2019)* | Y | Y | N | Y | N | N | N | N | N | N | Y | 4 |
| *Woo & Avery (2021)* | Y | Y | Y | Y | Y | Y | Y | Y | Y | Y | Y | 11 |
| *Zhang et al. (2024)* | Y | Y | Y | Y | Y | Y | N | Y | Y | Y | Y | 10 |

Y=Yes, N=No, U=Unclear, N/A=Not Applicable

- Yes and N/A = positive score
- No or Unclear = negative score
