# Supplementary material for: Healthcare workers’ sustainable employability in relation to quality of care: an umbrella review
Source: BMJ Open. 2025 Sep 8;15(9):e095126. doi: 10.1136/bmjopen-2024-095126 (PMC12421189; doi:10.1136/bmjopen-2024-095126)
Supplement: online supplemental file 3 [file bmjopen-15-9-s003.pdf]

Supplemental file 3: Characteristics of included studies (n = 55)

**Author/ year/ country**	**Objective(s)**	**Review Type & included designs**	**Participants and context setting**	**Databases, date range**	**# included studies, type of studies and country of origin**	**Quality appraisal methods + quality ratings**	**Relevant Exposure/ determinant / phenomenon of interest**	**(Relevant) Quality of Care Outcomes**
**Abraham et al. (2020)**<br><br>**US**	To identify studies investigating predictors and outcomes of burnout among Primary Care Providers' (PCPs) in the United States	Systematic review<br><br>Quantitative	Primary care providers in the United States	PubMed, Embase, CINAHL, Cochrane Library, Ovid Medline, ProQuest, Joanna Briggs Institute, PsychINFO<br><br>No set time frame	21 studies<br><br>19 cross-sectional, 2 cohort designs<br><br>US (21)	JBI critical appraisal checklists for cross-sectional and cohort studies<br><br>Studies had to receive a 'yes' for at least 5 out of 8 questions in the cross-sectional checklist, and a 'yes' for at least 7 out of 11 questions in the cohort studies checklist<br><br>All 21 studies were deemed of sufficiently quality to be included	Burnout	Quality of patient care, medical errors, likelihood of medical errors, patient satisfaction
**Al-Ghunaim et al. (2022)**<br><br>**UK/Saudi Arabia**	To conduct a systematic review and meta-analysis of studies that investigated the association between burnout and 1) patient safety and/or 2) surgical professionalism	Systematic review and Meta-Analysis of quantitative studies	Practicing and trainee surgeons (n=27248)	PsycINFO, Ovid MEDLINE(R), EMBASE, Cochrane Database, CINAHL, Web of Science<br><br>Until February 2021	14 studies (9 included in the meta-analysis)<br><br>United States (9), France (1), Germany (1), Greece (1), Poland (1), China (1)	Cochrane Risk of Bias Tool	Burnout (or sub facets, e.g. emotional exhaustion and depersonalization	Medical errors; involvement in patient safety incident patient satisfaction, malpractice suits, surgeons' perceived quality of care,
**Baatiema et al. (2017)**<br><br>**Ghana/Australia**	To identify health professionals' views on the barriers and enablers to their use of the above recommended evidence-based acute stroke care interventions.	Systematic review<br><br>Quantitative & Qualitative	Nurses, general medical doctors, neurologists, emergency department physicians, allied health	MEDLINE, CINAHL, Embase, PsycINFO, Cochrane Library, AMED	10 studies (3 qualitative, 7 quantitative)<br><br>Online and postal surveys, semi-structured interviews and focus group methods	Joanna Briggs Institute Checklist for assessing qualitative studies (10 items) & Centre for Evidence-Based management guidelines to appraise surveys (12 items)<br><br>Overall quality was moderate	Lack of skills, lack of knowledge	Evidence-based acute stroke care

			staff and health managers (n=1692)	1990-2016	Australia (4), USA (3), Sweden (2), Norway (1), Denmark (1), The Netherlands (1)			
**Balan (2021)**<br><br>**Malaysia**	To identify, critically evaluate, and summarize the findings on the knowledge, attitude and practice of Malaysian health care professionals towards adverse drug reaction (ADR) reporting.	Systematic review<br><br>Quantitative & Qualitative	Pharmacists, doctors, nurses in Malaysia	PubMed, Google Scholar, NMRR<br><br>January 2009 to December 2019	9 studies (2 qualitative, 7 quantitative)<br><br>Interviews, questionnaires	Critical appraisal of a questionnaire study (Roever, 2015); Critical Appraissal Skills Programme (CASP) checklist (2018) for qualitative research<br><br>5/9 studies were of high quality (= meeting 75% of indicators tools)	Insufficient clinical knowledge	Reporting of adverse drug reactions
**Basil et al. (2022)**<br><br>**Malaysia**	To estimate and critically appraise the evidence on the prevalence, causes and severity of medication administration errors amongst neonates in Neonatal Intensive Care Units (NICUS)	Systematic review & meta-analysis<br><br>Quantitative	Neonates in the NICU	PubMed, Embase, CINAHL, Web of Science, Scopus, Medline, PsycArticles, PsycInfo, International Pharmaceutical Abstracts, Google Scholar, Open Grey	20 studies<br><br>Direction observation technique, reviewing incident reports, patient charts<br><br>Egypt (5), India (3), Tunisia (2), Argentina, Brazil, Canada, Iran, Malaysia, Mexico, The Netherlands, Spain, Switzerland, USA (1)	A quality appraisal framework established by Allan and Barker (1990) for studies on medication errors<br><br>12 criteria, 12 points. 9-12 points: high quality; 5-8 points: moderate quality, below 5 points: low quality.<br><br>None met all 12 criteria, 1 study was rated as high quality, 15 as moderate quality, 4 as poor quality	Knowledge-based mistakes, fatigue	Medication Administration Errors
**Bromley et al. (2015)**<br><br>**USA/The Netherlands**	To provide a systematic review of the literature pertaining to barriers to colonoscopic screening in African Americans	Systematic review<br><br>Quantitative & Qualitative	Studies conducted in the US	MEDLINE, Cochrane Central Register of Controlled Trials<br><br>1950-November 2013	19 studies (8 qualitative, 11 quantitative<br><br>Interviews, focus groups, survey, chart review within randomized trial, retrospective review of claims,	Consolidated Criteria for Reporting Qualitative Research (COREQ)<br><br>Studies ranged from 16-25 out of 32 items (mean 19)<br><br>Strengthening the Reporting of Observational Studies in Epidemiology (STROBE)<br><br>Studies ranged from 16-21 out of 22 (mean 17)	Lack of guideline knowledge, lack of knowledge on patient barriers	Offering colonoscopy screening in African Americans
**Carey et al. (2019)**<br><br>**Australia**	To explore the types of barriers and enables to palliative care provision reported by primary care practitioners, and how commonly these are reported	Systematic review<br><br>Quantitative	Primary care practitioners, primary care doctors, specialist and primary care practitioners<br><br>Middle to high-income countries	Medline, Embase, PsychINFO<br><br>January 2007-March 2019	21 studies<br><br>Surveys<br><br>The Netherlands (5), Australia (4), United Kingdom (3), Canada (1), US (1), Austria (1), Switzerland (1), Finland (1), Denmark (1), Japan (1), Hong Kong (1), The Netherlands and Northern Ireland combined (1)	Joanna Briggs Institute Appraisal Checklist for studies reporting prevalence data<br><br>All studies had at least one high risk of bias or unclear judgement	(Lack of) skills, confidence, experience	Delivery of palliative care by primary care practitioners

**Chatfield et al. (2017)**<br><br>**USA**	To integrate findings from qualitative research reports on hand hygiene compliance among healthcare workers worldwide that employs the GRADE-CERQual process of quality assessment	Qualitative meta-summary<br><br>Qualitative	Health care workers	CINAHL, PubMed, PsycInfo, Healthsourc e/Nursing Academic, EMBASE, Sage complete<br><br>After 2001	36 studies<br><br>Interviews, focus groups, ethnographic, mixed method<br><br>Countries are not narratively reported	Modified version of the Critical Appraisal Skills Programme (rather than scoring yes/no/not certain for 10 items, the authors scored with 0-5 points for each item, allowing for more nuance<br><br>Maximum of 50 points. <25 points: poor or fair quality (17 studies), >25 points: good or better quality (19 studies)<br><br>Quality of individual studies is reported in a table	Lack of knowledge	Hand hygiene compliance rates
**Cho & Steege (2021)**<br><br>**USA**	To review the literature and to synthesize the evidence regarding the relationship between hospital nurse fatigue and nurse, patient, and organizational outcomes	Systematic review<br><br>Quantitative	Nurses who provide direct patient care in hospital settings	PubMed, PsychINFO, CINAHL, Plus, Scopus<br><br>January 2000-July 2020	22 studies<br><br>Cross-sectional (17), prospective longitudinal (4), retrospective longitudinal (1)<br><br>United States (6), Iran (5), Japan (2), Norway (2), Canada (1), Mainland China (1), Egypt (1), Lebanon (1), Oman (1), South Korea (1), Taiwan (1)	National Institutes of Health (NIH) Quality Assessment Tool for Observational Cohort and Cross-Sectional Studies (2017)<br><br>Poor quality (3), fair quality (18), good quality (1)	Work-related fatigue	Nurses' capacity to perceive patient deterioration cues; patient safety activities, patient safety management activities,
**Cormico n et al. (2023)**<br><br>**UK**	To identify health care professionals' perceived barriers and facilitators to clinical practice guideline implementation within stroke rehabilitation	Systematic review<br><br>Qualitative & quantitative	Health care professional working in any stroke rehabilitatio n setting	CINAHL, MEDLINE, PsycINFO, EMBASE, AMED, Cochrane Library, Academic Search Complete, Scopus<br><br>No date limit	22 studies<br><br>Designs are not summarized in text, but available in supplemental appendix<br><br>Australia, the Netherlands, Canada, Ireland, New Zealand, UK & Iceland	Mixed Method appraisal tool<br><br>Quality of individual studies is available in supplemental appendix<br><br>No studies were excluded based on quality	Competence (skills and knowledge)	Implementation of clinical practice guidelines within stroke rehabilitation
**Craig et al. (2016)**<br><br>**Australia**	To identify relevant barriers and enablers to six key clinical behaviours in acute stroke care	Systematic review using the Theoretical Domains Framework<br><br>Qualitative & Quantitative	Healthcare professional in the ED of a hospital	CINAHL, EMBASE, MEDLINE, Web of Science, PubMed Central, The Joanna Briggs Institute EBP, ProQuest Dissertation	9 studies (5 qualitative, 4 quantitative)<br><br>Interviews, focus groups, surveys<br><br>USA (4), Australia (2), Sweden (1), The Netherlands (1), France (1)	Critical Appraisal Skills Programme (CASP) for qualitative studies, the Centre for Evidence-Based Management "Appraisal of a Survey" tool for quantitative studies<br><br>Quality of individual studies is available in a table	Knowledge, skills	Six key clinical behaviours in acute stroke care

				s & Thesis, Agency for Healthcare Research and Quality, Open Grey, Grey Literature Report<br><br>No date limit				
**De Angelis et al. (2016)**<br><br>**Italy**	To describe and synthesise previous research on factors conditioning the spontaneous reporting of adverse drug reactions among nurses	Integrative review<br><br>Qualitative & Quantitative	Nurses	MEDLINE, Embase, CINAHL Plus, Scopus, Google scholar<br><br>January 2003-April 2014	16 studies (16 quantitative)<br><br>Observational, semi-experimental, quantitative-qualitative<br><br>Countries are summarized in a table	STROBE checklist (22 items) for cross-sectional studies; STROBE checklist (11 items) for conference abstracts; Quantitative critical appraisal tool (Law et al. 1998)<br><br>Scores per item with '+' or '-' amounting to a score from 0% to 100%. Scores: >80%: category A; between 50% and 80%: category B; <50%: category C.<br><br>Quality of the studies included were >50%. No studies were excluded based on methodological quality. Results for individual studies are available in online supplemental file	Knowledge	Spontaneous reporting of adverse drug reactions
**De Jong et al. (2016)**<br><br>**The Netherlands**	To investigate the relationship between Common Mental Disorders (CMDs) and medical incidents and/or unprofessional behaviour in hospital physicians	Systematic review<br><br>Quantitative	Hospital physicians	Medline (through PubMed)<br><br>2003-December 2013	15 studies<br><br>Cross-sectional (10), longitudinal (5)<br><br>USA (11), Europe (2), South Korea (1), Mexico (1)	Critical appraisal checklist similar to Gärtner et al. (2010) (8 items)<br><br>Scores per item with '+' or '- '. Total score is 8.<br><br>5 studies met all criteria, 9 met six or more, one study met 5 criteria.	Burnout, depression, fatigue, sleepiness	Quality of patient care, medical error,
**Dewa et al. (2017a)**<br><br>**USA/Canada**	To address the question, 'How does physician burnout affect the quality of healthcare related to the dimensions of acceptability and safety?'	Systematic review<br><br>Quantitative	Physicians practicing in civilian settings (and that have completed training)	MEDLINE Current, MEDLINE In-Process, MEDLINE Epub Ahead of Print, PsycINFO, Embase, Web of Science<br><br>Jan. 2002-Feb. 2017	12 studies<br><br>Designs not reported/summarized<br><br>USA (4), Germany (2), Greece (1), Israel (1), Japan (1), China (1), Taiwan (1), multinational study (Italy, Spain and Portugal) (1)	Nine-item risk of bias checklist developed based on the 'Strengthening of Observational Studies in Epidemiology criteria', recommendations by Sanderson et al. (2007) and Lagerveld et al. (2010).<br><br>'1' indicated met criteria, maximum score of '9'. 1-4 points is considered high risk of bias, 5-7 points moderate risk of bias and 8-9 points low risk of bias<br><br>10/12: moderate risk of bias<br>2/12: high risk of bias	Burnout	Medical errors; patient satisfaction; perceived general quality of care

**Dewa et al. (2017b)**<br><br>**USA/Canada**	To address the question, "How does resident burnout affect the quality of healthcare related to the dimensions of acceptability and safety?"	Systematic review<br><br>Quantitative	Residents working in civilian settings	Medline Current, Medline In-Process, PsychINFO, Embase, Web of Science<br><br>Jan 2002 – September 2015	10 studies<br><br>Designs not reported/summarized<br><br>US (8), The Netherlands (1), Mexico (1)	Nine-item risk of bias checklist developed based on the 'Strengthening of Observational Studies in Epidemiology criteria', recommendations by Sanderson et al. (2007) and Lagerveld et al. (2010).<br><br>'1' indicated met criteria, maximum score of '9'. 1-4 points is considered high risk of bias, 5-7 points moderate risk of bias and 8-9 points low risk of bias<br><br>7/10: moderate risk of bias<br>3/10: high risk of bias	Burnout	Medical error; suboptimal care
**Egerton et al. (2017)**<br><br>**Australia**	To identify and synthesize available qualitative evidence on primary care clinicians' views on providing recommended management of osteoarthritis	Systematic review/evidence synthesis<br><br>Qualitative	Primary care clinicians	MEDLINE, Cochrane Central Register of Controlled Trials, Embase, CINAHL and PsycInfo<br><br>Up until 30 August 2016	8 studies<br><br>Study designs are not narratively reported<br><br>Australia, (2), France (2), UK (2), Germany, Mexico	Critical Appraisal Skills Programme (CASP) checklist<br><br>Quality of individual studies is available in a table	Lack of knowledge, feeling under-prepared	Management of osteoarthritis in line with clinical practice guidelines
**Fathizadeh et al. (2024)**<br><br>**Iran**	To investigate prevalence of medication errors and the associated factors among Iranian nurses	Systematic review & meta-analysis<br><br>Quantitative	Iranian nurses	PubMed, Web of Science, Scopus, Google Scholar, IranMedex, Magiran, SID<br><br>No date limit	36 studies (6,238 participants)<br><br>Designs or countries are not narratively reported/summarized	Critical appraisal checklist for cross-sectional studies (Joanna Briggs Institute (JBI)<br><br>8 items: >6 is high quality, 3-6 is moderate quality, <3 is low quality<br><br>Quality of individual studies is not narratively summarized, but available in a table	Fatigue	Medication error
**Fekonja et al. (2023)**<br><br>**Slovenia**	To identify and examine the factors contributing to patient safety during the triage process	Systematic review<br><br>Quantitative, Qualitative, & Mixed-method studies	Triage nurses working in the ER department	PubMed, CINAHL, Web of Science, Science Direct, SAGE, EMBASE	11 studies (6 qualitative, 5 quantitative)<br><br>Focus groups, interviews, observation, questionnaires, assessment tool, review of medical records<br><br>US (3), Sweden (3), Iran (1), Taiwan (1), Australia (1), South Korea (1), Canada (1)	JBI Critical Appraisal Checklist for 1) qualitative research and for 2) analytical cross-sectional research<br><br>Low quality (60-69%); medium quality (70-79%); high quality (80-89%); excellent quality (more than 90%). Minimal requirements for inclusion were a score of 60 or more.<br><br>7 studies (moderate quality), 1 study (high quality), 3 studies (excellent quality).	Skills/knowledge, burnout, fatigue	Patient safety during triage in the emergency department

						One study was eventually excluded because of insufficient quality.		
**Gallione et al. (2022)**<br><br>**Italy**	To explore and synthesize the available literature on extrinsic and intrinsic factors acting as barriers or facilitators in nurses' implementation of Clinical Practice Guidelines (CPGs)	Systematic review<br><br>Quantitative, qualitative & mixed-method studies	Nurses	MEDLINE, Embase, CINAHL<br><br>January 2010 – February 2021	60 studies (16 qualitative, 34 quantitative, 10 mixed method)<br><br>RCT, observational, survey, mixed-method, focus groups, interviews, content-analysis, implementation projects, ethnography,<br><br>No information on studies' country of origin	Mixed Method Appraisal Tool (MMAT)<br><br>Outcome of quality appraisal is not summarized in text	Knowledge and skills	Implementation of Clinical Practice Guidelines (CPGs)
**Gates et al. (2018)**<br><br>**Canada**	To synthesize evidence on the (1) impacts of insufficient sleep and fatigue on health and performance, and patient safety and (2) effectiveness of interventions targeting insufficient sleep and fatigue.	Systematic review and meta-analysis<br><br>Quantitative & Qualitative	Physicians (residents excluded) in independent practice within a high-income country (to mirror Canadian setting) (n=36190)	Medline, Embase, PsycINFO, CINAHL and PubMed<br><br>2000-2016 (Medline was updated to November 2017)	47 studies (all quantitative)<br><br>observational (45), intervention studies (2)<br><br>6 were pooled in meta-analysis<br><br>North America (20); Europe (16); other (11)	Cochrane Risk of Bias Tool (RCT)<br><br>Effective Practice and Organization of Care Group (adaptation) (before/after + time-series)<br><br>Newcastle Ottawa Quality Assessment Scale (cohort) and adapted version (cross-section, non-comparative)<br><br>Overall quality was poor, 62% unclear or high risk of bias.	Fatigue, insufficient sleep	Perceived quality of care, self-perceived medical errors, patient outcomes (e.g. intraoperative complications, postoperative complications, mortality, length of stay)
**Hall et al. (2016)**<br><br>**UK**	To explore the association between wellbeing, burnout and wellbeing & burnout together in relation to patient safety in healthcare professionals.	Systematic review<br><br>Quantitative	Health care professionals that directly deal with patients	PsycINFO, MEDLINE, Embase, Scopus<br><br>No restrictions on year of publication	46 studies<br><br>cross-sectional (33), prospective (9), other (4)<br><br>16 different countries, most from America (19)	COCHRANE risk of bias tool and additional questions<br><br>Individual quality is not reported, overall quality on appraisal items is reported but not scored.	Burnout, mental ill-health	Errors
**Hamed & Konstantinidis (2022)**<br><br>**UK**	To identify barriers to incident reporting among nurses	Systematic review<br><br>Qualitative	Nurses	PubMed, CINAHL, HMIC, PsycINFO, EMBASE<br><br>2000 until 2022	5 studies (qualitative)<br><br>Focus groups (2), interviews (2), interview + email (1)<br><br>Iran (2), Korea (1), Malaysia (1), China (1)	Critical Appraisal Skills Program (CASP) for qualitative studies<br><br>Overall, quality was relatively good	Lack of knowledge/skills	Incident reporting
**Hodkinson et al. (2022)**<br><br>**UK/Greece**	To examine the association between physician burnout with the career engagement and the quality of patient care globally	Systematic review & meta-analysis<br><br>Quantitative	Physicians	Medline, PsycINFO, Embase, CINAHL<br><br>Up until May 2021	170 studies (quantitative)<br><br>Study designs are not summarized<br><br>US (77), Europe (48), UK (4), African region (2), Region of the America (8), South-East Asian Region (2), Eastern Mediterranean	Newcastle Ottawa Critical Appraissal Tool<br><br>Max. score is 7, with 0-2 classified as high risk of bias, 3-5 as medium risk, and 6-7 as low risk<br><br>Overall, 32 (19%) of studies reported low risk of bias, 115 (367%) reported medium risk of bias, and 23 (14%) reported high risk of bias	Burnout	Patient safety incidents (prescribing errors, potentially avoidable readmission, prescribing errors, monitoring errors,

					Region (3), Western pacific Region (29), multi-national study (1)			and potentially avoidable adverse events); Patient satisfaction
**Houghton et al. (2020)**<br><br>**Ireland/Norway/UK**	To identify barriers and facilitators to healthcare workers' adherence to IPC guidelines for respiratory infectious diseases.	A rapid qualitative evidence synthesis<br><br>Qualitative, mixed methods (with qualitative component)	Health care workers	Ovid Medline<br><br>No year limit	Initial screening phase yielded 36 studies, but due to the nature of the review a representative sample was used of 20 studies<br><br>Interviews (9), focus groups (8) Interviews and focus groups (2), focus groups and textual data from questionnaire (1), focus groups and nominal group technique after focus groups (1)<br><br>Australia (2), Canada (2), Hong Kong (1), Singapore (2), South Korea (2), Taiwan (2), USA (1), China (1), the Dominican Republic (1), India (1), Russia (1), South Africa (3), Uganda (1)	Adapted version of the Critical Skills Appraisal Programme. Evidence assessed using the GRADE-CERQual approach.<br><br>Over 50% of the included studies had no or minor methodological limitations. Six studies had moderate or major limitations. 3/26 findings were graded as high confidence, 18 as moderate, 5 as low	Knowledge	Adherence to Infection Prevention and Control (IPC) guidelines for respiratory infectious diseases
**Janes et al. (2021)**<br><br>**UK**	To assess the relationship between staff engagement and patient safety outcomes and explore if they way these variables were defined and measured has any differential effect	Systematic review and meta-analysis<br><br>Quantitative	Health care staff	Medline, CINAHL, PsycInfo, Embase, Cochrane Library, National Institute for Health Research Journals<br><br>January 1999-February 2020	14 studies (11 included in the meta-analysis)<br><br>Cross-sectional (13), non-cross-sectional (1)<br><br>North America/Canada (7), Europe (4), Middle/Far East (3)	Guidance on the assessment of observational studies (Thomas et al., 2004), Cochrane risk of bias tool plus a priori team-defined criteria<br><br>Scores ranged from 0-6, 5-6 being high, 3-4 being fair, 0-2 being poor quality<br><br>Quality appraisal: high quality (2), fair quality (7), poor quality (5)	Engagement	Patient safety culture, number of errors/adverse events
**Jun et al. (2016)**<br><br>**USA**	To appraise and synthesize the current literature on barriers to and facilitators in the use of clinical practice guidelines (CPGS) by registered nurses	Integrative review<br><br>Qualitative & Quantitative	Registered nurses	PubMed, CINAHL, ProQuest, Medline, Embase<br><br>1995-2015	16 studies (7 quantitative, 9 qualitative)<br><br>Cross-sectional surveys, qualitative descriptive studies, grounded theory, phenomenology<br><br>USA (4), Australia (2), Canada (2), Finland (1), Singapore (1), Sweden (2), the Netherlands (4)	Critical Appraisal Skills Program for cohort (12 items) and qualitative studies (10 items)<br><br>Answer items: yes (2 points)/no (0 points)/can't tell (1 points)<br><br>Quality of individual studies is reported in a table. One article as labelled low quality, but was nonetheless included	Knowledge	Use of clinical practice guidelines

**Jun et al. (2021)**<br><br>**USA**	To systematically and critically appraise the current literature to examine the associations between nurse burnout and patient and hospital organizational outcomes	Systematic review<br><br>Quantitative	Nurses in hospital settings	PubMed, CINAHL, PsycInfo, Scopus, Embase<br><br>No data restrictions	20 studies<br><br>Cross-sectional<br><br>United States (6), Belgium (3), Canada (2), Taiwan (3), Brazil (2), Ecuador (1), Germany (1), Italy (1), Iran (1), Japan (1), New Zealand (1), Switzerland (1), The United Kingdom (1), Thailand (1) (This includes one study covering six different countries)	Critical Appraisals Programme (CASP) for cohort studies<br><br>Maximum score: 24<br><br>Quality appraisal scores ranged from 17 to 22.5, mean score was 20.7 indicating moderate quality	Burnout	Perceived patient safety, nurses' reporting behaviours, mortality, patient falls, medication error, nurses' perceived quality of care, patients' perceived quality of care, urinary tract infection, surgical site infection, nurse adherence to infection control precautions, patient experience, patient satisfaction
**Kelly et al. (2023)**<br><br>**Ireland**	To identify and describe the most frequently reported enablers and barriers to implementing (inter)national endorsed standards, in order to inform the selection of strategies that can optimise their implementation	Systematic review and meta-summary<br><br>Quantitative, qualitative, and mixed method	Standards in health and social care	Medline, CIAHL, SocINDEX, Google Scholar, OpenGrey, GreyNet International<br><br>Up until November 2021	35 studies (from 37 reports) (21 quantitative, 9 qualitative, 5 mixed method)<br><br>Questionnaires, focus groups, interviews, mixed method (focus groups, interviews, observation, and surveys)<br><br>USA (9), Australia (8), UK (5), The Netherlands (4), Iran (2), Bangladesh/Brazil/Croatia/Ethiopia, Jordan/Republic of South Africa/and WHO regions (including 180 countries) (1)	Critical Appraisal Skills Programme Tool for qualitative studies; Jonna Briggs Institute Critical Appraisal Tools for quantitative studies; The Mixed Methods Appraisal Tool for mixed methods studies; Evidence-based Librarianship critical appraisal checklist for one group post-test design<br><br>19 studies: no methodological limitations; 14 had minor methodological limitations; 4 had moderate methodological limitations.	(lack of) Knowledge	Implementation of (inter)nationally endorsed health and social care standards
**Keyko et al. (2016)**<br><br>**Canada**	To determine wat is currently known about the antecedents and outcomes of work engagement in professional nursing practice	Systematic review<br><br>Qualitative & Quantitative	Nursing practice	CINAHL, MEDLINE, PsycINFO, PROQUEST, SCOPUS, Web of Science, EMBASE, Business Source Complete<br><br>No date limits	18 studies (1 qualitative, 2 mixed-methods, 15 quantitative)<br><br>Designs are not reported narratively<br><br>Canada (7), USA (7), Australia (1), China (1), Iran (1), Taiwan (1)	For correlational studies, the reviewers used a quality appraisal tool that multiple other reviews (13 criteria, with a total of 14 points)<br><br>0-4 points: low quality; 5-9 points: moderate quality; 10-14 points: high quality<br><br>An intervention study was appraised using a pre/post quality assessment tool adapted from another systematic review<br><br>Qualitative research was appraised with Critical Skills Appraisal Programme<br><br>No studies were excluded based on quality. Results: 17 studies were rated as moderate or	Work engagement	Voice behaviour, perceived care quality, quality care, patient satisfaction, adverse events

						high quality; the intervention study as low quality and the rest can be found in a table.		
**McFadden et al. (2020)**<br><br>**UK/India/Switzerland**	To examine facilitators of, and barriers to, providing high-quality midwifery care in India	Systematic review<br><br>Quantitative & Qualitative	Pregnant women, postnatal mothers, women of childbearing age, health service providers in India	MIDIRS, MEDLINE, MEDLINE in Process, CINAHL, CDSR, HTA database, Social Science Citation Index, PsycINFO, HMIC, ASSIA, Social Policy and Practice, British Nursing Index, Research Councils UK, OAIster<br><br>No limit on year of publication	32 studies (25 quantitative, 6 qualitative, 1 mixed methods)<br><br>Surveys, service evaluations, interviews, focus groups	For quantitative studies: appraising selection bias, study design, data collection methods and confounding. Assessments ranged from low risk, high risk and unsure treat as high risk for particular domains. Mixed method is both appraised in the quantitative and the qualitative instrument (hence the different number of studies)<br><br>12/26 studies: low risk of bias across all domains; 14/26 studies: low risk selection bias; 26/26 studies low risk study design; 17/26 studies: low risk data collection; 20/26 studies: low risk for confounding<br><br>Qualitative studies: categorizing studies as 'strong' or weak based on assessing 'clear exposition of methods of data generation and analysis, triangulation of data, respondent validation, and reflexivity.<br><br>2/6 studies: 'strong'; 4/6 studies: 'weak'	Lack of knowledge, skills and competence of skilled birth attendants	Provision of high-quality midwifery care
**Ng et al. (2021)**<br><br>**Australia**	To explore the barriers and enablers that influence the application of a biopsychosocial approach to musculoskeletal pain in practice, from a clinicians' perspective	Systematic review and qualitative evidence synthesis<br><br>Qualitative	Primary healthcare professionals	MEDLINE, PsycINFO, EMBASE, CINAHL, Scopus<br><br>No date limit	25 studies<br><br>1 dissertation, 24 qualitative studies<br><br>(Australia (1), Canada (5), Germany (3), Ireland (1), Italy (1), The Netherlands (1), New Zealand (2), Sweden (1), UK (7), United States (2), Brazil (1).	Critical Appraisal Skills Programme (CASP) Checklist in combination with the Consolidated Criteria for reporting qualitative research (COREQ) checklist.<br><br>No studies would be excluded based on methodological quality. Three possible quality ratings: low, moderate and high.<br><br>14 studies (high quality), 6 studies (moderate quality), 5 studies (low quality)	Knowledge/skills	Adoption of a biopsychosocial approach to musculoskeletal pain
**Niño de Guzmán et al. (2020)**<br><br>**Spain/Italy/Chile/Peru**	To assess the extent of healthcare providers' adherence to breast cancer CGs in Europe and to identify the factors that impact on healthcare providers' adherence.	Systematic review<br><br>Quantitative & qualitative	Health care providers in European countries	MEDLINE, Embase<br><br>Until May 2019	41 studies (reported in 57 references)<br><br>Retrospective cohort-design (19), cross-sectional (13), non-controlled before-after studies (5); prospective cohort (4); case study (1), qualitative study (1)	AXIS tool (cross-sectional; range 0-10; 1-4 (low), 5-7 (moderate, 8-10 (high); Quality Assessment Tool for Before-After (Pre-Post)Studies with No Control Group (non-controlled before and after studies; good, fair or poor quality); Newcastle-Ottawa Quality Assessment Scale (prospective and retrospective cohort studies; range 0 (=highest risk of bias to 9 (lowest risk of bias); Critical	Lack of knowledge	Health care providers' adherence to breast cancer Clinical Guidelines (CGs)

					The Netherlands (13); Italy (8); other European countries (20)	Appraisal Skills Programme checklist (qualitative)<br><br>40/41 studies were appraised (case-study was not considered suitable for quality appraisal). 25/40 studies: low risk of bias; 14/40 studies: moderate risk of bias; 1/40 studies: high risk of bias.		
**O'rorke et al. (2022)**<br><br>**USA**	To guide the maturation of current communication skills curricula, we performed a systematic review to examine the aspects of surgeon communication that have a positive impact on patient satisfaction.	Systematic review<br><br>Qualitative	Surgeons	PubMed, Embase, Scopus, Ovid/Medline<br><br>From January 1, 2000, onwards	26 studies (qualitative)<br><br>Countries or designs are not narratively reported	Modified version of the Critical Appraisal Skills Program (CASP) to appraise qualitative studies<br><br>Quality of studies is not narratively reported, but available in a table.	Communication and interpersonal skills	Patient satisfaction, patient outcomes
**Okuyama et al. (2014)**<br><br>**Japan/The Netherlands**	To 1) assess the effectiveness of speaking up for patient safety, 2) to evaluate the effectiveness of speaking up training, 3) to identify the influencing factors of speaking-up behaviour by health care professionals, and 4) to develop a model for health care professionals' speaking-up behaviour by integrating these factors into the model of employee speaking up behaviour.	Systematic review<br><br>Quantitative & Qualitative	Hospital-based physicians, medical residents, fellows, nurses	PubMed, MEDLINE, CINAHL, Web of Science, the Cochrane Library<br><br>Until December 2012	21 studies (reported in 27 articles)<br><br>Interviews, surveys,<br><br>US (19), UK (3), US & Japan (1)	Authors appraised quality with 5 criteria: aims and objectives are clearly stated, the design is clearly specified and appropriate for the aims of the research; researchers provide a clear account of the process by which their findings were reproduced; researchers display enough data to support their interpretations and conclusions, and the method of analysis was appropriate and adequately executed.<br><br>Everything was scored good, only a few studies provided limited data to support conclusions	Job satisfaction, knowledge	Speaking-up behaviour
**Parajuli & Hupcey (2021)**<br><br>**USA**	To synthesize the evidence on the barriers to palliative care utilization in an oncology population	Systematic review<br><br>Qualitative, Quantitative and Mixed methods	Oncology clinicians	PubMed, CINAHL, PsycInfo<br><br>2008 to May 2020	29 studies (8 quantitative, 18 qualitative, 3 mixed-methods studies)<br><br>Designs are not narratively reported<br><br>US (9), Australia (6), Belgium (2), Europe (2), Canada (2), Germany (1), Japan (1), India (1), Iran (1), Turkey (1), Columbia (1), France (1), France/Belgium (1)	Mixed Method Appraisal Tool (MMAT) (5 items)<br><br>3 response options: yes/no/can't tell<br><br>Overall, 16 studies (all qualitative) met all criteria); the remainder did not meet 2 or more criteria. One mixed-method study had none of the criteria met<br><br>Quality of individual studies are available in a table	Providers' lack of knowledge about PC, communication skills	palliative care utilization in an oncology population

**Parry et al. (2015)**<br><br>**UK**	To examine international research relating to the contributing factors to registered nurses' behaviour that result in a medication administration error event.	Narrative review<br><br>Quantitative	Registered nurses working in countries with developed healthcare systems, in acute adult hospital patient settings	Cochrane, MEDLINE, CINAHL, BNI, Embase, PsycInfo<br><br>Jan. 1999- December 2012	26 studies<br><br>Self-report or direct observation<br><br>United States/Canada (13), Europe (4), Australia (4), Asia (4), multi-national study (1)	STROBE reporting guidelines for observational research<br><br>High quality: 11 studies<br>Moderate quality: 15 studies<br>Low quality: 1 (was excluded)	Clinical expertise (assessed as knowledge/abilities), fatigue/tiredness/sleepiness, job satisfaction	Medication administration error event
**Peng et al. (2023)**<br><br>**China/Japan**	To discover the factors impacting on missed nursing care of nurses	Qualitative meta-synthesis<br><br>Qualitative	Nurses	PubMed, Web of Science, Cochran Library, CINAHL, MEDLINE, ScienceDirect<br><br>No date range limit	9 studies (8 qualitative, 1 mixed methods)<br><br>Australia (3), Iran (2), Italy (1), Ireland (1), Israel (1), Singapore (1)	Joanna Briggs Institute Qualitative Assessment and Review Instrument (QARI)<br><br>No study was excluded due to poor quality, but quality of included studies is not narratively summarized	Dissatisfaction with the career	Missed Nursing Care (MSC)
**Pereira-Lima et a. (2019)**<br><br>**USA/Brazil**	To provide summary relative risk (RR) estimates for the associations between physician depressive symptoms and medical errors.	Systematic review & meta-analysis<br><br>Quantitative	Physicians & residents (	Embase, ERIC, Pubmed, PsycInfo, Scopus, Web of Science<br><br>Until 2018	11 studies (21517 physicians)<br><br>longitudinal (7), cross-sectional (4)<br><br>USA (9), Japan (1), South Korea (1)	Adapted criteria from the Cochrane Library guidelines<br><br>6 studies considered as methodologically strong on the basis of design, 8 on the basis of ascertainment of depressive symptoms measure, 8 on the representativeness of the sample; all considered strong on the basis of descriptive characteristics of participants.	Depression	Medical errors
**Pitzer et al. (2024)**<br><br>**Austria/ Germany**	To review the current evidence on barriers that impair, delay, or prohibit access to palliative care for adult hospital inpatients	Mixed-methods systematic review<br><br>Quantitative Qualitative, Mixed methods	Healthcare professional	MEDLINE, CINAHL, PsycINFO, Cochrane Library<br><br>October 2003- December 2020	79 studies (49 quantitative, 27 qualitative, 3 mixed method)<br><br>Retrospective chart review, semi structured interviews, surveys, cohort study, focus groups, qualitative observation, in-person interviews, prospective observational study, rapid ethnographic assessment<br><br>USA (45), UK (8), Australia (7), Taiwan (4), Japan (3), Canda (2), France (2), India (1), Brazil (1), Sweden (1), The Netherlands (1), Germany (1), Tanzania (1), Ghana (1), Nigeria (1)	QualSyst systematic review tool. Provides two ratings cales, one for quantitative and one for qualitative methods<br><br>Scores range from 0.0 to 1.00<br>Low quality studies were not excluded<br>Overall mean quality rating was 0.76, with studies ranging between 0.29 to 1.00. Quality of individual studies is available in an appendix.	(insufficient) knowledge about palliative care	Access to palliative care

**Putri et al. (2024)**<br><br>**Indonesia**	To investigate healthcare professionals' knowledge, awareness, attitude and practice on pharmacovigilance and adverse drug reaction reporting, explore the causes of the underreporting issue, and provide improvement strategies	Systematic review<br><br>Quantitative	Health care professional s'	PubMed, Scopus, Google Scholar, Scholar ID<br><br>1st of January 2012 – 31st of December 2022	25 studies<br><br>Surveys<br><br>Saudi Arabia, India, South Africa, Nepal, Pakistan, Bosnia & Herzegovina, Southwestern Nigeria, Vietnam, Jordan, Turkey, Ghana, and Indonesia	Joanna Briggs Institute Critical Appraisal Checklist Tools for analytical cross-sectional studies<br><br>Answer options: yes/no/unclear/not applicable<br><br>All studies scored >50% 'yes' on items in the checklist. Details for individual studies can be found in an appendix.	Knowledge	Adverse drug reaction reporting
**Reijmeri nk et al. (2024)**<br><br>**The Netherla nds**	To assess the effect of non-muscular fatigue on surgical outcome	Systematic review<br><br>Quantitative	Qualified surgeons and surgical residents	MEDLINE, Embase<br><br>Up until 17 January 2023	134 studies	The Cochrane risk of bias tool for RCTs and the Newcastle-Ottawa scale for cohort studies<br><br>Poor quality cohort studies or studies scoring at high risk of bias in any of the 6 bias domains were excluded<br><br>Quality of individual studies is not reported	Fatigue	Surgical outcome in real-life studies
**Salmasi et al. (2016)**<br><br>**Malaysia**	To identify and review studies on the incidence and types of Medication Errors (MEs) in Southeast Asian countries in order to identify common MEs and estimate its prevalence in this region	Systematic review<br><br>Quantitative	Southeast Asian countries (Brunei, Cambodia, Indonesia, Laos, Malaysia, Myanmar, Philippines, Singapore, Thailand, Timor and Vietnam	PubMed, Embase, Medline, Proquest Central, CINAHL plus	17 studies<br><br>Survey, observational (direct, prospective), retrospective study, cohort, descriptive<br><br>Singapore (5), Malaysia (4), Thailand (3), Vietnam (2), Philippines (1), Indonesia 1, Brunei/Laos/Cambodia/Myanmar/T imor (0)	Thirteen criteria adapted from Alsulami et al. (2013).<br><br>Total scores either represented poor, average and good quality<br><br>59% were of poor or average quality, 41% were of good quality	Lack of knowledge	Medication errors
**Salyers et al. (2017)**<br><br>**USA**	To systematically review and quantify empirical studies linking healthcare provider burnout to quality and safety in order to better understand the magnitude and consistency of these relationships. We explored potential moderators to examine whether the relationships would vary as a function of the aspect of burnout or quality being studied.	Meta-analysis<br><br>Quantitative	Healthcare providers	Ovid MEDLINE, PsycInfo, Web of Science, CINAHL, and ProQuest Dissertation s & Theses<br><br>Until March 2015	102 studies (82 unique samples of health care providers (n=210,669)<br><br>designs not summarized/reported<br><br>North America (42), Europe (31), Asia (5), Australia (2), Africa (1), South America (1)	Cochrane Collaboration's Tool for assessing risk of bias in randomised trials; Tool from the National Collaborating Centre for Methods and Tools and other literature on assessing bias (13 items in total).<br><br>Reported in only supplementary table. No overall ratings reported.	Burnout	Perceived quality, patient satisfaction, perceived safety, safety events

**Scheepers et al. (2015)**<br><br>**The Netherlands/USA**	To study the effect of physicians' occupational well-being on the quality of patient care.	Systematic review<br><br>Quantitative	Physicians	MEDLINE, Embase, PsycInfo<br><br>Until August 2014	18 studies<br><br>cross-sectional (17), pre-post test (1)<br><br>USA (9), Germany (1), Spain (1), The Netherlands (1), other European countries (2), UK (1), Japan (1), Taiwan (1), Australia (1)	Medical Education Research Study Quality (MRSQI) (score can range from 5 – 18)<br><br>Quality ranged between 6.5 and 13, average quality 9.8	Occupational well-being: measured as job satisfaction (14 studies), career satisfaction (3), work engagement (1)	Medical errors, Avoidant/superfluous care, patient satisfaction, patient adherence to treatment, suboptimal care, self-perceived quality of care, patients' self-reported pain and depressive symptoms
**Schroers et al. (2021)**<br><br>**USA**	To critique and synthesize the qualitative evidence on perceived causes of Medication Administration Errors (MAEs) as reported by nurses in health settings	Systematic review<br><br>Qualitative	Nurses	CINAHL, PubMed, Scopus, Google Scholar<br><br>2000-February 2019	16 studies<br><br>Semi-structured interviews, focus groups, self-administered questionnaire, direct observation, review of narratives of case reports, ethnography, informal conversations<br><br>UK (6), and 8 other countries	Critical Appraissal Skills Programme (CASP)<br><br>Legitimacy of the research process (all 16 studies), confirmability (all 16 studies), credibility and dependability (varied between studies)	Lack of medication knowledge	Medication Administration Error (MAE)
**Slade et al. (2016)**<br><br>**Australia/Denmark/UK**	To perform a systematic review and metasynthesis of qualitative studies that have explored the perceptions and beliefs of primary care clinicians about Low Back Pain (LBP) Clinical Practice Guidelines (CPGs), including perceived enablers and barriers to guideline adherence.	Systematic review and metasynthesis<br><br>Qualitative	Primary care clinicians who commonly manage LBP (e.g., general medical practitioners, PTs, chiropractors, osteopaths, occupational therapists)	MEDLINE, EMBASE, The Cochrane Central Register of Controlled Trials, AMED, PsychInfo, SportDiscus, EBM Reviews<br><br>Until July 2014	17 studies<br><br>Focus groups, questionnaire with open-ended questions, interviews<br><br>UK (5), Canada (4), The Netherlands (2), USA (2), Germany (1), Israel (1), New Zealand (1), Norway (1).	Critical Appraissal Skills Programme (CASP) checklist for qualitative studies<br><br>Around 50% of studies did not meet the twelve quality items considered to be important for trustworthiness	Minimal knowledge of the existence of guidelines or their content, and how they were derived; lack of confidence in the ability to assess LBP and provide evidence-based care	Use of LBP CPGs
**Smiddy et al. (2015)**<br><br>**Ireland**	To gain a better understanding of the issues that affect health care workers' compliance with hygiene guidelines from a qualitative perspective.	Systematic review<br><br>Qualitative	Health care workers	MEDLINE, Embase, CINAHL,	10 studies<br><br>interviews (7), focus groups (2), interviews and focus groups (1)<br><br>Canada (3), UK (2), Australia (2), NL (1), US (1), Taiwan (1)	Critical Appraisal Skills Programme for qualitative research and additional methodological recommendations for quality appraisal<br><br>All but one study showed methodological weaknesses (common: lack of a theoretical framework, author reflexivity, inadequate	Knowledge	Compliance with hand hygiene guidelines

				2000-2014 (June)		information on interpretation and analysis of the data)		
**Tawfik et al. (2019)**<br><br>**USA/UK**	To estimate the overall relationship between burnout and quality of care and to evaluate whether published studies provide exaggerated estimates of this relationship.	Systematic review & meta-analysis<br><br>Quantitative	Health care provider	MEDLINE, PsychINFO, Health and Psychosocial Instruments (EBSCO), Mental Measurements Yearbook (EBSCO), EMBASE, Web of Science<br><br>Until 2019 (May 28)	123 studies (142 distinct study populations, n= 241553 health care providers)<br><br>Cross-sectional, observational, longitudinal<br><br>Countries of origin are not summarized/reported (but available in online supplement)	Authors do not conduct 'traditional' quality appraisal, but correct individual studies' effect sizes for several biases using statistical methods/tools. Biases tested for: small sample bias, correlation bias (by selecting only one first-listed/primary outcome), excess significance,<br><br>Excess significance was observed for adherence to best practice guidelines and for quality and safety metrics. However, excess significance may be a result of genuine heterogeneity of effects across studies rather than reporting bias. Studies using independent or objective quality metrics demonstrated less frequent significant effects. There was no evidence for small-study effects.	Burnout	Inappropriate labs, inappropriate timing of discharge, suboptimal patient care practices, inappropriate use of patient restraints, inappropriate antibiotic prescribing, lack of close monitoring, neglect of work, forgetting to convey information, not fully discussing treatment options, poor handoff quality, diagnosis delay, poor pain control, low patient enablement score, poor adherence to infection control, poor adherence to management guidelines, self-reported medical errors, observed medical errors, diagnosis errors, adverse events, self-reported medication error, self-reported treatment/medication errors, observed medication errors, health care associated infections, patient falls, length of stay, urinary tract infections, mortality, morbidity, post-hospitalization

								recovery time, prolonged emergency department visit, near-miss reporting, safety perceptions, quality during most recent shift, quality of care, safety climate score, malpractice allegations
**Thomas et al. (2019)**<br><br>**Qatar/UK**	To critically appraise, synthesize, and present the evidence of medication errors amongst hospitalized patients in Middle Eastern countries, specifically prevalence, nature, severity and contributory factors	Systematic review<br><br>Qualitative and quantitative	Hospital settings in the Middle East (Bahrain, Egypt, Iran, Iraq, Israel, Jordan, Kuwait, Lebanon, Oman, Palestine, Qatar, Saudi Arabia, Syria, Turkey, United Arab Emirates, Yemen	CINAHL, Embase, Medline, PubMed, Science Direct<br><br>From 2000-end of March 2018	50 studies (48 quantitative, 2 qualitative)<br><br>Questionnaires, incident reporting systems, direct observation of practice, semi-structured interviews, patient medical records<br><br>Iran (23), Saudi Arabia (10), Egypt (5), Jordan (5), Turkey (2), Israel (1), Qatar (1), Yemen (1), Palestine (1), Lebanon (1)	STROBE checklist, adapted to a quality assessment tool (11 items)<br><br>13 studies: scored 8 or more items; 21 studies: scored between 5 and 7; 16 studies: 4 or less.<br><br>Quality of individual studies is available in a supplementary table.	Lack of knowledge	Medication error
**Toomey et al. (2021)**<br><br>**Australia/Singapore**	To identify determinants (barriers and facilitators) of eye care delivery by optometrists and interventions that may improve eye care delivery	Systematic review<br><br>Qualitative, quantitative, mixed methods	Optometrists, ophthalmic opticians	PubMed, MEDLINE, EMBASE, CINAHL, SCOPUS, PsycINFO, ProQuest, Web of Science	30 studies (21 quantitative, 5 mixed methods, 4 qualitative)<br><br>Study designs are not reported in text/table<br><br>Australia (11), UK (8), Canada (4), USA (3), India (2), New Zealand (2), Saudi Arabia (1), Ireland (1), Ghana (1)	Mixed Methods Appraisal Tool (MMAT)<br><br>No studies were excluded due to low quality<br><br>10/30 studies: extremely high quality (meeting all criteria)<br><br>Quality of individual studies is available in a figure	Knowledge, skills	Eye care delivery (in line with evidence-based practice)
**Vaismoradi et al. (2020)**<br><br>**Norway/Spain/UK/Finland**	To understand factors that influence disclosing and reporting practice errors by nurses in residential long-term care settings	Systematic review<br><br>Qualitative & Quantitative	Nurses in residential long-term care settings	PubMed, Medline, Scopus, CINAHL, Embase<br><br>2010-2019	5 studies (2 quantitative, 2 qualitative, 1 mixed methods)<br><br>Survey, prospective cohort study, content analysis, thematic analysis, mixed methods<br><br>Canada (1), Czech Republic (1), Norway (2), USA (1)	STROBE for cross-sectional, observational and cohort studies (max. score 34)<br><br>2 studies: one scoring 22, another scoring 24<br><br>COREQ for qualitative research (max. score 32)<br><br>2 studies: one scoring 21, another scoring 24	Knowledge	Disclosing and reporting practice errors

						GRAMMS for mixed-methods studies (max. score 12)<br><br>1 study: scoring 9		
**Vrbnjak et al. (2016)**<br><br>**Slovenia/ Ireland**	To explore barriers to nurses' reporting of medication errors and near misses in hospital settings	Systematic review<br><br>Qualitative, Quantitative, Mixed methods	Nurses in hospital settings	Medline, CINAHL, PubMed, Cochrane Library<br><br>No date limit	38 studies (30 quantitative, 5 qualitative, 3 mixed methods)<br><br>Questionnaires, interviews, focus groups, or a combination of the former (mixed methods)<br><br>US (12), Iran (5), Taiwan (3), UK (3), Jordan (3), Saudi Arabia (2), Canada (2), Australia (2), Egypt (1), Israel (1), South Korea (1), Nigeria (1), Malta (1)	Mixed Methods Appraisal Tool (MMAT) (19 items)<br><br>Scores between 0% and 100%, with 0%: very low, 25%: low, 50%: moderate, 75%: high: 100%: very high<br><br>Very low-quality studies were excluded (1). Moreover, 4 studies were considered low quality, 14 studies as moderate quality, 13 studies as high quality, and 7 studies as very high quality. Scores of individual studies are listed in a table	Knowledge, skills	Reporting medication errors and near misses
**Wee & Lai (2022)**<br><br>**USA**	To provide a precise estimate of the strength of the relationship between work engagement among health care professionals and quality of care to better inform research and practice on workforce well-being	Systematic review & Meta-Analysis<br><br>Quantitative	Health care professionals providing direct patient care	ABI Inform, BIOSIS, EBSCO, OpenGrey, ProQuest Dissertations and Theses, PsychINFO, PubMed, ScienceDirect, Scopus, Web of Science<br><br>No restriction on publication date	25 studies<br><br>Cross-sectional, longitudinal, or mix of both<br><br>13 countries across 4 continents, spanning 132,664 health care providers	Joanna Briggs Institute's (JBI) critical appraisal checklist for cross-sectional studies and cohort studies<br><br>To be included, there was a minimum of 5 (out of 8) questions for the cross-sectional checklist and at least 7 (out of 11) questions for the cohort checklist<br><br>All included studies were deemed of sufficient quality to be included	Work engagement	Quality of care (overall/unspecified), objective or subjective quality of care, patient safety, patient perceived quality of care, nurse perceived quality of care, quality of care, 7-day patient mortality, quality of care,
**Woo & Avery (2021)**<br><br>**Singapore/Australia**	To examine and understand nurses' experiences of voluntary error reporting (VER) and elucidate factors underlying their decision to engage in VER	Integrative literature review<br><br>Quantitative & Qualitative	Nurses	CINHAL, Ebsco, Medline (PubMed), Scopus, Embase<br><br>Jan. 2010-Jan. 2020	31 studies (21 quantitative, 9 qualitative, 1 mixed methods)<br><br>Cross-sectional, semi-structured interviews<br><br>Countries are not reported narratively	Mixed Methods Appraisal Tool (MMAT)<br><br>Two papers were eliminated due to low quality. Quality of individual studies is not narratively summarized, but available in a table.	Deficiency in knowledge	Voluntary error reporting (VER)

**Zhang et al. (2024)**<br><br>**China/Australia**	To conduct a systematic review to synthesize the updated empirical studies on physician empathy and its effects on patient outcomes and doctor-patient communication	Systematic review<br><br>Qualitative, quantitative and mixed method	Physicians	Web of Science, MEDLINE, Scopus<br><br>2017-2021	11 studies (7 quantitative, 2 qualitative, 2 mixed-method studies)<br><br>Study designs are not reported narratively<br><br>USA (4), NL (3), Norway (3), UK (2), France (1), Canada (1), China (1)	Mixed Method Apppraisal Tool (MMAT)<br><br>Quality appraisal is presented in a table rather than narrative form	Physician empathy as a communicative competence	Patient functional status, patient satisfaction
