# Supplementary material for: Healthcare workers’ sustainable employability in relation to quality of care: an umbrella review
Source: BMJ Open. 2025 Sep 8;15(9):e095126. doi: 10.1136/bmjopen-2024-095126 (PMC12421189; doi:10.1136/bmjopen-2024-095126)
Supplement: online supplemental file 4 [file bmjopen-15-9-s004.docx]

| **Supplemental file 4: Extracted findings** | | | | | | |
| --- | --- | --- | --- | --- | --- | --- |
| **Table A. Health indicators of SE in relation to QoC outcomes** | | | | | | |
| **BURNOUT** | | | | | | |
| **Author (year)** | **Determinant** | | **Quality of Care** | **Indicator of SE** | **Results** | **Direction of (majority of) evidence** |
| Abraham et al. (2020) | Primary care provider burnout | | Quality of patient care (perceived by providers) | Perceived health status | One study included found no association between PCP burnout and quality of patient care | No relation |
|  | Primary care provider burnout | | Suboptimal patient care | Perceived health status | One study found an association between PCP burnout and suboptimal patient care | Positive relation |
|  | Primary care provider burnout | | (likelihood of) medical error | Perceived health status | One study found no association between PCP burnout and medical errors, whilst another study found that PCP burnout was associated with an increase in the likelihood of medical error | Mixed evidence |
|  | Primary care provider burnout | | Patient satisfaction | Perceived health status | One study found that PCP (specifically physicians) burnout was not associated with differences in patient satisfaction | No relation |
| Al-Ghunaim et al. (2022) | Surgeon burnout | | Medical errors | Perceived health status | Higher burnout is significantly associated with a higher risk of involvement in a medical error | Partial relation, depends on the sub facet of the burnout measure. |
|  | Surgeon depersonalization (sub facet of burnout) | | Medical errors | Perceived health status | Depersonalization in surgeons is not significantly associated to medical errors |  |
|  | Surgeon burnout | | Patient satisfaction | Perceived health status | Burnout is not significantly associated with patient satisfaction | No relation |
|  | Surgeon burnout | | Malpractice suits | Perceived health status | Burnout is strongly related to malpractice suits | Positive relation |
|  | Surgeon burnout | | Perceived quality of safety (as perceived by surgeons) | Perceived health status | Burnout is significantly associated with poorer perceived quality of care among male, but not among female surgeons | Partial relation |
|  | Surgeon emotional exhaustion (sub facet of burnout) | | Patient safety incident | Perceived health status | Higher emotional exhaustion levels in surgeons are significantly positively associated to a greater likelihood of being involved in a patient safety incident | Positive relation |
| De Jong et al. (2016) | | Resident burnout score | Perceived quality of patient care | Perceived health status | One study found that residents with high burnout scores were significantly less likely to report good quality of patient care | Negative relation |
|  |  | Physician/resident burnout | Medical error | Perceived health status | One study found that medical errors were significantly related to all three domains of burnout (emotional exhaustion, depersonalization, decreased personal accomplishment). Another study corroborates these findings for two domains, but not for ‘personal accomplishment’ (not significant). Three other studies found a significant association between self-reported errors and burnout. One longitudinal study, however, found similar rates of errors between burnout out residents and non-burned-out residents | Positive relation |
|  |  | Junior doctor burnout | Suboptimal patient practices | Perceived health status | One study found that junior doctors with burnout were 5.5 times more likely to report one or more suboptimal patient practices monthly (significant), and 5.2 times more likely to report such practices weekly (significant). | Positive relation |
|  |  | Physician burnout | Patient satisfaction | Perceived health status | One study found a significant negative correlation between burnout (all three domains) and patient satisfaction with dialysis care. Another study, however, did not find a significant association between physician burnout and patients’ satisfaction, confidence or trust in their physicians. | Mixed evidence |
| Dewa et al. (2017a) | | Physician burnout | Medical errors (self-perceived) | Perceived health status | There is a consistent significant positive relationship between physician burnout and medical errors among four papers | Partial relation, only when medical errors are self-perceived rather than based on chart audits |
|  |  | Physician burnout | Medical errors (based on chart audits) | Perceived health status | The relationship between physician burnout and medical errors – based on chart audits – is not significant |  |
|  |  | Physician burnout (or at least one dimension of burnout) | Patient satisfaction | Perceived health status | There is a consistent and significant relationship between physician burnout (or at least one dimension of burnout) and patient satisfaction. Especially high depersonalization (sub facet of burnout) is consistently significantly related to lower patient satisfaction. The significance of emotional exhaustion (sub facet of burnout) is not consistent, however. | Partial relation, depends on the subscale of the burnout measure |
|  |  | Physician burnout (or at least one dimension of burnout) | Quality of care (as perceived by physicians) | Perceived health status | There is a consistent and significant relationship between physician burnout (or at least one dimension of burnout) and self-perceived quality of care. Looking at separate dimensions of burnout, emotional exhaustion was consistently significantly related to self-perceived quality of care, but depersonalization was not. | Partial relation, depends on the subscale/dimension of the burnout measure |
| Dewa et al. (2017b) | | Resident burnout | Medical errors (self-perceived) | Perceived health status | There is a consistent significant positive relationship between resident burnout and self-perceived medical errors across studies | Partial relation, only when medical errors are self-perceived rather than based on chart audits. |
|  |  | Resident burnout | Medical errors (reviews of clinical records) | Perceived health status | One study finds that resident burnout is no longer significantly associated to medical errors if these are assessed by reviewing clinical records |  |
|  |  | Resident depersonalization (sub facet of burnout) | Suboptimal patient care practices | Perceived health status | Depersonalization (sub facet of resident burnout) is significantly positively related to suboptimal patient care practiced monthly or weekly. | Positive relation |
| Hall et al. (2016) | | Burnout | Errors | Perceived health status | 21/30 studies found that healthcare practitioner burnout is significantly associated to making more errors. 4/30 found partial associations (with subscales of either burnout or safety measures). 5/30 did not find any significant associations between burnout and error. | Positive relation |
| Fekonja et al. (2023) | | Nurse burnout | Patient safety during triage in the emergency department | Perceived health status | Triage nurses indicated burnout as a major impediment to safe and effective triage of patients | Negative relation |
| Hodkinson et al. (2022) | | Physician burnout | Patient safety incidents (prescribing errors, potentially avoidable readmission, prescribing errors, monitoring errors, and potentially avoidable adverse events)  *(Classified as safety events in the overview of evidence table)* | Perceived health status | Physician burnout was significantly associated with double the risk of patient safety incidents compared with no patient safety incidents based on overall burnout score, but also emotional exhaustion, depersonalization, and reduced personal accomplishment (sub measures of burnout). | Positive relation |
|  |  | Physician burnout | Patient satisfaction | Perceived health status | Physician burnout was also significantly associated with up to threefold decreases in patient satisfaction compared with patients being satisfied based on measures of overall burnout, but also depersonalization and reduced personal accomplishment. | Negative relation |
| Jun et al. (2021) | | Nurses’ emotional exhaustion (sub facet of burnout) | Perceived patient safety | Perceived health status | Nurses’ emotional exhaustion is consistently negatively associated with perceived patient safety | Negative relation |
|  |  | Nurses’ emotional exhaustion (sub facet of burnout) | Nurses’ reporting behaviours | Perceived health status | Nurses’ emotional exhaustion is consistently negatively associated with nurses’ reporting behaviours | Partial relation, depends on the sub facet of the burnout measure |
|  |  | Nurses’ personal achievement (sub facet of burnout) | Nurses’ reporting behaviours | Perceived health status | Nurses’ personal achievement is not consistently associated with nurses’ safety reporting behaviours |  |
|  |  | Nurses’ emotional exhaustion (sub facet of burnout) | Mortality | Perceived health status | One study found that higher emotional exhaustion is a significant predictor for increased mortality | Positive relation |
|  |  | Nurses’ depersonalization (sub facet of burnout) | Patient falls | Perceived health status | Nurses’ depersonalization was associated with increased nurse-reported adverse events, such as falls |  |
|  |  | Nurse burnout | Urinary tract infection | Perceived health status | Nurse burnout is significantly associated with increased rate of urinary tract infection |  |
|  |  | Nurse burnout | Surgical site infection | Perceived health status | Nurse burnout is significantly associated with increased rate of surgical site infections |  |
|  |  | Nurses’ depersonalization (sub facet of burnout) | Medication errors | Perceived health status | Nurses’ depersonalization was associated with increased nurse-reported adverse events, such as medication errors. | Positive relation |
|  |  | Nurse burnout (all three sub facet) | Nurses’ perceived quality of care | Perceived health status | All three subscales of burnout were significantly correlated with poor/fair quality of care as assessed by nurses | Negative relation |
|  |  | Nurse burnout | Patients’ perceived quality of care | Perceived health status | Burnout is not significantly correlated with quality of care as assessed by patients | No relation |
|  |  | Nurse burnout | Nurse adherence to infection control precautions | Perceived health status | Nurse burnout was found to be a negative predictor of nurses’ adherence to infection control precautions | Negative relation |
|  |  | Nurse burnout | Patient experience | Perceived health status | Nurse burnout is negatively associated with patient experiences | Negative relation |
|  |  | Nurses’ emotional exhaustion (sub facet of burnout) | Patient satisfaction | Perceived health status | Nurses’ emotional exhaustion is negatively associated with patient satisfaction |  |
| Salyers et al. (2016) | | Burnout | Provider reported perceived quality of care | Perceived health status | There is a significant negative relationship between burnout and provider reported quality of care | Negative relation |
|  |  | Burnout | Patient satisfaction | Perceived health status | There is a significant negative relationship between burnout and patient reported quality of care (measured as patient satisfaction) | Negative relation |
|  |  | Burnout | Safety (measured as providers’ perceptions of safety and errors) | Perceived health status | There is a significant negative relationship between burnout and safety | Negative relation. In the overview of evidence table, we have coded this as a positive relation (i.e., ‘green’) between burnout and errors (i.e. more errors is decreased safety), and as a negative relation between burnout and providers’ perceptions of safety (lower perceived safety) |
| Tawfik et al. (2019) | | Burnout | Inappropriate labs  *(Categorized under ‘suboptimal care’)* | Perceived health status | Positive relation (small effect size) (according to Hedges g estimates) | Positive relation |
|  |  | Burnout | Inappropriate timing of discharge  *(Categorized under ‘suboptimal care’)* | Perceived health status | Positive relation (medium-high effect size) (according to Hedges g estimates) | Positive relation |
|  |  | Burnout overall, and all three dimensions of burnout | Suboptimal patient care practices | Perceived health status | Positive relation (medium-high effect size) (according to Hedges g estimates) | Positive relation |
|  |  | Burnout | Inappropriate use of patient restraints  *(Categorized under ‘suboptimal care’)* | Perceived health status | Positive relation (small effect size) (according to Hedges g estimates) | Positive relation |
|  |  | Emotional exhaustion, depersonalization, low personal accomplishment | Inappropriate antibiotic prescribing  *(Categorized under ‘suboptimal care’)* | Perceived health status | Positive relation for emotional exhaustion and low personal accomplishment (small effect size) (according to Hedges g estimates), negative relation for depersonalization (small effect size) (according to Hedges g estimates) | Partial relation, depending on the sub facet of burnout measure used |
|  |  | Burnout | Lack of close monitoring  *(Categorized under ‘suboptimal care’)* | Perceived health status | Positive relation (small effect size) (according to Hedges g estimates) | Positive relation |
|  |  | Burnout | Neglect of work  *(categorized under ‘missed care’)* | Perceived health status | Positive relation (high effect size) (according to Hedges g estimates) |  |
|  |  | Burnout | Forgetting to convey information  *(Categorized under ‘missed care’)* | Perceived health status | Positive relation (small effect size) (according to Hedges g estimates) |  |
|  |  | Burnout | Not fully discussing treatment options  *(Categorized under ‘suboptimal care’)* | Perceived health status | Positive relation (moderate effect size) (according to Hedges g estimates) |  |
|  |  | Burnout | Poor handoff quality  *(Categorized under ‘suboptimal care’)* | Perceived health status | Positive relation (small effect size) (according to Hedges g estimates) |  |
|  |  | Burnout | Diagnosis delay  *(Categorized under ‘suboptimal care’)* | Perceived health status | Negative relation (small effect size) (according to Hedges g estimates) | Negative relation |
|  |  | Burnout overall, and emotional exhaustion and low personal achievement | Poor pain control  *(Categorized under ‘suboptimal care’)* | Perceived health status | Burnout overall and low personal achievement are positively related to poor pain control (small effect size), whilst emotional exhaustion is negatively related to poor pain control (small effect size) | Partial relation, depending on the sub facet of burnout measure |
|  |  | Emotional exhaustion, depersonalization, low personal accomplishment | Low patient enablement score | Perceived health status | Emotional exhaustion and low personal accomplishment have a negative relation (small effect size according to Hedges g estimates) with low patient enablement score and depersonalization also has a negative relation (moderate effect size according to Hedges g estimates) | Negative relation (i.e. less low patient enablement, in other words: better patient enablement, hence why it is reversely coded as a positive relation (i.e., ‘green’) in the overview of evidence table. |
|  |  | Burnout | Poor adherence to infection control | Perceived health status | Positive relation (small effect size) (according to Hedges g estimates) | Positive relation, i.e. more poor adherence to infection control, in other words: less adherence to infection control, hence why it is reversely coded as a negative relation (i.e., ‘red’) in the overview of evidence table |
|  |  | Emotional exhaustion and Depersonalization | Poor adherence to management guidelines | Perceived health status | Positive relation (medium-high effect size) (according to Hedges g estimates) |  |
|  |  | Burnout overall, and all three dimensions of burnout | Medical errors (self-reported) | Perceived health status | Positive relation (small effect size) (according to Hedges g estimates) | Positive relation |
|  |  | Burnout | Observed medical errors | Perceived health status | Positive relation (small effect size) (according to Hedges g estimates) |  |
|  |  | Burnout | Diagnosis errors | Perceived health status | Positive relation (small effect size) (according to Hedges g estimates) |  |
|  |  | Burnout overall, and all three dimensions of burnout | Adverse events | Perceived health status | All positive relation, but small effect for burnout overall, and depersonalization and low personal accomplishment, while large effect for emotional exhaustion |  |
|  |  | Burnout overall, and all three dimensions of burnout | Medication error (self-reported) | Perceived health status | Positive relation (small effect size) (according to Hedges g estimates) | Positive relation |
|  |  | Burnout overall, and all three dimensions of burnout | Treatment/medication errors (self-reported | Perceived health status | Positive relation with burnout (overall) and depersonalization and low personal accomplishment (moderate effect size according to Hedges g estimates), and emotional exhaustion (small effect size according to Hedges g estimates) |  |
|  |  | Burnout | Observed medication error | Perceived health status | Positive relation (large effect size) (according to Hedges g estimates) |  |
|  |  | All three dimensions of burnout | Healthcare associated infections | Perceived health status | All positive (small effect size) | Positive relation |
|  |  | All three dimensions of burnout | Patient falls | Perceived health status | All positive (small effect size) | Positive relation |
|  |  | Burnout overall, and all three dimensions of burnout | Length of stay | Perceived health status | Burnout overall is negatively related to length of stay (moderate effect size), whilst all three dimensions are positively related to length of stay (small effect size) | Partial relation, depending on how burnout is measured (overall or via subdimensions) |
|  |  | All three dimensions of burnout | Urinary tract infections | Perceived health status | All positive (small effect size) | Positive relation |
|  |  | All three dimensions of burnout | Mortality | Perceived health status | Depersonalization and low personal accomplishment are positively related with a small effect size, emotional exhaustion is positively related with moderate effect size | Positive relation |
|  |  | Emotional exhaustion | Morbidity | Perceived health status | Positively related, small effect size | Positive relation |
|  |  | All three dimensions of burnout | Post-hospitalization recovery time | Perceived health status | Emotional exhaustion and low personal accomplishment are negatively related to post-hospitalization, whilst depersonalization is positively related to post-hospitalization recovery time (all small effect sizes) | Partial relation, depending on the sub facet of burnout measure |
|  |  | Burnout overall | Prolonged emergency department visit | Perceived health status | Positive relation (small effect size) (according to Hedges g estimates) | Positive relation |
|  |  | Emotional exhaustion and depersonalization | Near-miss reporting | Perceived health status | Emotional exhaustion is negatively related to near-miss reporting (moderate effect size), and depersonalization is positively related to near-miss reporting (moderate effect size) | Partial relation, depending on the sub facet of burnout measure |
|  |  | Emotional exhaustion and depersonalization | Low safety perceptions | Perceived health status | Both positively related, but emotional exhaustion large effect size, and depersonalization small effect size | Positive relation, i.e. more low safety perceptions, in other words: provider perceived safety has decreased, hence why it is reversely coded as a negative relation (i.e., ‘red’) in the overview of evidence table |
|  |  | Emotional exhaustion and depersonalization | Low quality (as perceived by HCWs) during most recent shift | Perceived health status | Both positively related (moderate effect size) | Positive relation, i.e. more low perceived quality, in other words: provider perceived quality of care is decreased, hence why it is reversely coded ‘i.e. ‘red’) in the overview of evidence table |
|  |  | Burnout overall, and all three dimensions of burnout | Low quality of care  *(categorized as ‘aggregated/unspecified quality of care’)* | Perceived health status | All positively related, but depersonalization as a small effect size and the rest as a moderate effect size | Positive relation, i.e., more low quality of care, in other words: quality of care has decreased, hence why it is reversely coded (i.e., ‘red’) in the overview of evidence table |
|  |  | Burnout overall, and all three dimensions of burnout | Low safety climate score | Perceived health status | Burnout overall, low personal accomplishment and depersonalization have a positive relation (respectively large, moderate and small effect size), and emotional exhaustion has a negative relation (small effect size) | Partial relation, depending on the (sub facet) of burnout measure |
|  |  | Burnout overall, and all three dimensions of burnout | Malpractice allegations | Perceived health status | All positively related (small effect size) | Positive relation |
| **MENTAL ILL-HEALTH** | | | | | | |
| De Jong et al. (2016) | | Resident/physician depression | Medical error | Perceived health status | One study found a significant association between resident depression and errors occurring often or multiple times. Another study also found that depressed residents were significantly more likely to report a medical error compared with non-depressed residents. Three other studies also report that the relationship between depression and medical errors yielded odds ratios between 2.21 and 3.29. | Positive relation |
|  |  | Resident depression | Medication error | Perceived health status | One study found a significant positive relationship between resident burnout and medication errors. | Positive relation |
| Hall et al. (2016) | | Wellbeing | Errors | Perceived health status  *(Wellbeing in this study is measured as mental health, distress, depression, anxiety, and job stress’, as such we have categorized it under the indicator 'mental ill-health'* | 16/27 studies found that poor wellbeing (measured as mental health, distress, depression, anxiety, and job stress) was associated with poorer patient safety (measured as error). 6/27 studies corroborate this finding, but only for a few subscales of the wellbeing measures or safety measures. 5/27 studies found no correlation between wellbeing and patient safety (measured as error) | Positive relation |
| Pereira-Lima et al. (2019) | | Physician/resident depressive symptoms | Perceived medical errors | Perceived health status | 11 studies involving 21517 physicians demonstrated an association between physician depressive symptoms and an increased risk for perceived medical errors. The magnitude of the association did not differ much between residents and physicians. Longitudinal studies found a lower summary RR estimate compared with cross-sectional studies in the meta-analysis. Some sub-group analyses (non-US versus US, or non-surgical versus surgical specialty) yielded lower summary RR estimates, but all remained statistically significant. | Positive relation |
| **FATIGUE** | | | | | | |
| Basil et al. (2022) | | Fatigue | Medication administration error | Fatigue | Fatigue is one of the examples of individual-related factors contributing to medication administration errors | Positive relation |
| Cho & Steege (2021) | | Nurse work-related fatigue | Patient safety management activities | Fatigue | Nurse work-related fatigue is significantly negatively associated with patient safety management activities. | Negative relation |
|  |  | Nurse work-related fatigue | Nurse perception of patient safety | Fatigue | Nurse work-related fatigue is significantly negatively associated with nurses’ overall perceptions of patient safety, but another study included did not find a significant association between nurse work-related fatigue to nurses’ overall perceptions of patient safety | Mixed evidence |
|  |  | Nurse work-related fatigue | Self-reported nursing errors | Fatigue | Higher fatigue is significantly related to more frequent self-reporting nursing errors | Positive relation |
|  |  | Nurse work-related fatigue | Patient safety events reported | Fatigue | Higher fatigue is significantly associated with a lower frequency of events reported | Negative relation |
| De Jong et al. (2016) | | Resident/physician fatigue | Medical errors | Fatigue | Two studies describe a significant positive relationship between fatigue and medical errors. Another study did not find a significant relationship but did find that fatigue was a common contributing factor to medical error | Positive relation |
|  |  | Resident/physician sleepiness | Medical errors | Need for recovery | Three studies report a significant positive association between sleepiness and medical errors. | Positive relation |
| Fathizadeh et al. (2024) | | Nurse fatigue | Medication errors | Fatigue | Fatigue was identified as one of the three main causes for medication errors, accounting for 42,7% | Positive relation |
| Fekonja et al. (2023) | | Nurse fatigue | Patient safety during triage in the emergency department | Fatigue | Triage nurses have pointed out that a 12h-workday affects their decision-making and increases fatigue, and thus, patient safety according to them. | Negative relation |
| Gates et al. (2018) | | Physician fatigue | Perceived quality of care | Fatigue | A study of 890 physicians from Israel demonstrated that perceived quality of care was significantly predicted by fatigue, even after controlling for components of burnout | Negative relation |
|  |  | Fatigue or insufficient sleep | Medical errors (self-reported) | Fatigue/need-for-recovery | Five cross-sectional studies reported on self-reported errors, and these showed mixed findings for associations with fatigue or insufficient sleep. | Mixed evidence |
|  |  | Sleep-deprived surgeons | Mortality and postoperative complications | Fatigue/need-for-recovery | Meta-analyses of 6 studies showed that there is no difference in the rate of postoperative complications, nor patient mortality between sleep-deprived versus non-sleep deprived surgeons. | No relation |
|  |  | Sleep-deprived surgeons | Length of stay | Fatigue/need-for-recovery | One study on cardiac surgery, favoured sleep deprived surgeons over non-sleep deprived surgeons in terms of length of stay. However, other studies had null results. | No relation |
|  |  | Sleep-deprived surgeons | Intraoperative complications | Fatigue/need-for-recovery | For intraoperative complications, the findings of one study favoured non-sleep deprived surgeons, but the others had null results | No relation |
| Parry et al. (2015) | | Tired/exhausted/  sleepiness/ fatigue (nurses) | Medication administration error | Fatigue/Need for recovery | Three studies found that being tired or exhausted was viewed as a cause of medication administration error. Another study found a significant association between excessive daytime sleepiness and medication administration errors. Another study also reported fatigue as contributing to MAE. | Positive relation |
| Reiimerink et al. (2024) | | Fatigue (surgeons) | Surgical outcome in real-life studies | Fatigue | 35,4% of real-life studies report a deterioration in surgical outcome when fatigue was present. 8,2% of real-life studies reported an improvement in surgical outcome when fatigue was present. 8,2% of real-life studies report a mixed effect, meaning that both a deteriorating and improvement was observed when fatigue was present. 48,2% of real-life studies reported no discernible effect on surgical outcome when fatigue was present. | Mixed evidence |
| Schroers et al. (2021) | | Fatigue/physical exhaustion (nurses) | Medication Administration Errors | Fatigue | Nurses report that fatigue and physical exhaustion contribute to medication administration errors. | Positive relation |

| **Table B. Wellbeing indicators of SE in relation to QoC outcomes** | | | | | |
| --- | --- | --- | --- | --- | --- |
| **WORK ENGAGEMENT** | | | | | |
| **Author (year)** | **Determinant** | **Quality of Care** | **Indicator of SE** | **Results** | **Direction of (majority of) evidence** |
| Janes et al. (2021) | Staff engagement | Patient Safety Culture | Motivation | 9/10 studies reported positive, statistically significant correlations between staff engagement and patient safety. 7 studies suitable for meta-analysis showed a small positive, significant association | Positive relation |
|  | Staff engagement | Errors/adverse events | Motivation | Pooled analysis of 4 (out of 7) studies measuring the number of errors/adverse events indicated that higher engagement had a small, inverse relationship with error/events (r | Negative relation |
| Keyko et al. (2016) | Nurse work engagement | Voice behaviour | Motivation | Voice behaviour was reported to significantly increase with greater work engagement | Positive relation |
|  | Nurse work engagement | Perceived care quality | Motivation | Perceived care quality was reported to significantly increase with greater work engagement | Positive relation |
| Wee & Lai (2022) | Work engagement | Quality of care (overall/unspecified) | Motivation | Most of the studies (64%) found a significant and positive relationship between frontline health care professionals’ work engagement and patient quality of care. Eight studies did not find any significant relationship, and one study found a significantly negative relationship.  Meta-analysis indicates a significantly positive relationship between work engagement and quality of care, suggesting that a higher work engagement was associated with enhanced patient quality of care. The strength of the relationship is small to medium (per Cohen). | Positive relation |
|  | Work engagement | Quality of care (measured as either patient safety (unspecified) or quality of care (unspecified) | Motivation | The relationship between work engagement of health care providers and quality of care remains significant regardless of the type of the quality-of-care measure. | Positive relation |
|  | Work engagement | Quality of care (measured through subjective or objective measures) | Motivation | Two studies used objective sources for the quality-of-care measure, while fifteen studies used subjective self-assessments. The relationship between work engagement and quality of care was significantly positive for subjective self-assessment, whereas for objective data, this relationship is significantly negative. | Positive relation between quality of care (subjectively measured) and work engagement |
|  |  |  |  |  | Negative relation between quality of care (objectively measured) and work engagement |
|  | Work engagement | Quality of care (perceived by patients) | Motivation | 2/3 studies measured quality of care as perceived by patients found no significant association, another study did find a significant and positive relationship. | No relation |
|  | Work engagement (nurses versus physicians) | Quality of care | Motivation | For both professions, there is a significant positive relationship, but the difference between groups is not significant. | Positive relation |
|  | Work engagement | 7-day patient mortality | Motivation | One study found that job engagement was associated with lower 7-day patient mortality, but only for nurses, not for physicians. | Partial relation |
|  | Work engagement (measured with different scales) | Quality of care | Motivation | The relationship between work engagement and patient quality of care was significant regardless of work engagement scale used. | Positive relation |
| **JOB SATISFACTION** | | | | | |
| Okuyama et al. (2014) | Job satisfaction | Speaking-up behaviour | Job satisfaction | Persons who positively voice their concerns are generally more satisfied with their workplace and exhibit more discretionary efforts to speak up. | Positive relation |
| Parry et al. (2015) | Nurses job dissatisfaction | Medication administration error | Job satisfaction | Two studies found that registered nurses who were less satisfied with their work perceived higher frequencies of medication administration error | Positive relation, i.e., if job dissatisfaction of nurses increases, so do the medication errors. In other words, if nurses’ job satisfaction increases, then medication administration errors will likely decrease, hence why this relation is reversely coded (i.e., ‘red’) in the overview of evidence table |
| Peng et al. (2023) | Career dissatisfaction | Missed Nursing Care | Job satisfaction | Dissatisfaction with the career was reported as a reason for missed nursing care | Positive relation, i.e., if career dissatisfaction increases, so does missed nursing care. In other words, if nurses’ career satisfaction increases, then missed nursing care will likely decrease, hence why this relation is reversely coded (i.e., ‘red’) in the overview of evidence table. |
| Scheepers et al. (2015) | Physicians’ job satisfaction/work engagement | Medical errors | Job satisfaction | Physicians with higher levels of occupational well-being (measured as either job satisfaction or work engagement) reported fewer medical errors in two studies. However, two other studies on the same relationship do not report an association | Mixed evidence |
|  | Physicians’ job satisfaction | Avoidant or superfluous care  *(categorized under ‘suboptimal/missed care’)* | Job satisfaction | Physicians’ job satisfaction is not associated with avoidant or superfluous medical care in consultations | No relation |
|  | Physicians’ job satisfaction/career satisfaction | Patient satisfaction | Job satisfaction | Five studies showed a positive association between physicians’ job or career satisfaction and patient satisfaction in various specialties. One study reported no association between job satisfaction and patient satisfaction. | Positive relation |
|  | Physicians’ job satisfaction | Patient adherence to treatment | Job satisfaction | One study on patient adherence showed that patients of satisfied physicians adhered better to recommended medication, exercise, and diet than patients of physicians who were dissatisfied with their work | Positive relation |
|  | Physician’s job satisfaction | Suboptimal care | Job satisfaction | Physicians with higher levels of job satisfaction reported less suboptimal care | Negative relation |
|  | Physician’s job/career satisfaction | Self-perceived quality of care | Job satisfaction | Two studies showed that satisfied physicians reported better patient care quality than physicians who were less satisfied | Positive relation |
|  | Physician’s job satisfaction | Patients’ self-reported pain and depressive symptoms  *(Categorized under ‘patient outcomes’)* | Job satisfaction | Job satisfaction of physicians is not associated with patients’ self-reported pain and depressive symptoms | No relation |

| **Table C. Employability indicators of SE in relation to QoC outcomes** | | | | | |
| --- | --- | --- | --- | --- | --- |
| **KNOWLEDGE/SKILLS** | | | | | |
| **Author (year)** | **Determinant** | **Quality of Care** | **Indicator of SE** | **Results** | **Direction of (majority of) evidence** |
| Baatiema et al. (2017) | Lack of knowledge and/or skills | Adoption of evidence-based therapies for treatment and management of acute stroke | Skill-gap | Lack of skills and/or knowledge to apply or to use evidence-based interventions is identified as a barrier to evidence-based acute stroke care | Negative relation |
| Balan (2021) | Insufficient clinical knowledge | Adverse drug reaction reporting | Skill-gap | Most Malaysian healthcare professionals demonstrate good knowledge on ADR reporting, but insufficient clinical knowledge (classified under ‘lethargy’) is considered a barrier to ADR reporting. | Negative relation |
| Basil et al. (2022) | Lack of medical or medication knowledge | Medication administration error | Skill-gap | Knowledge-based mistakes, such as a lack of medical or medication knowledge, were frequently found as factors associated with medication administration errors | Positive relation |
| Bromley et al. (2015) | Lack of knowledge of guidelines | Offering colonoscopy in African Americans | Skill-gap | Poor provider knowledge regarding the age threshold (age: 45) for screening African Americans (in line with recommendations of the American College of Gastroenterology) is a barrier to offering colonoscopy screening in African Americans | Negative relation |
|  | Lack of knowledge of patient barriers |  |  | Poor provider knowledge on current patient barriers for screening of African Americans is a barrier to offering colonoscopy screening in African Americans |  |
| Carey et al. (2019) | Lack of confidence in managing specific symptoms | Delivery of palliative care  *(categorized under ‘(sub)optimal care’, with the delivery of palliative care being seen as optimal care)* | Skill-gap | Lack of confidence in managing specific symptoms is a barrier to delivering palliative care by primary care practitioners (PCPs) | Negative relation, i.e., lack of skills/knowledge/confidence on various tasks leads to less delivery of palliative care. In other words, it leads to more suboptimal care, hence why it is reversely coded (i.e., ‘green’) In the overview of evidence table. |
|  | Lack of confidence with dealing with psychological/social aspects for patient and/or family |  |  | Lack of confidence dealing with psychological/social aspects for patient and/or family is a barrier to the delivery of palliative care by PCPs |  |
|  | Lack of skills/confidence with treatments medications, or home care technology |  |  | Lack of skills/confidence with treatments medications, or home care technology is a barrier to the delivery of palliative care by PCPs |  |
|  | Lack of confidence with communication |  |  | Lack of confidence with communication is a barrier to the delivery of palliative care by PCPs |  |
|  | Lack of confidence dealing with PC emergencies |  |  | Lack of confidence dealing with PC emergencies is a barrier to the delivery of palliative care by PCPs |  |
|  | Lack of confidence with coordination of health care networks/services |  |  | Lack of confidence with coordination of health care networks/services is a barrier to the delivery of palliative care by PCPs |  |
|  | PCP knowledge, education, and experience in pain and symptom management |  |  | PCP knowledge, education, and experience in pain and symptom management is an enabler to the delivery of palliative care by PCPs | Positive relation |
|  | PCP knowledge, education, and experience in communication and psychosocial issues |  |  | PCP knowledge, education, and experience in communication and psychosocial issues is an enabler to the delivery of palliative care by PCPs. |  |
|  | PCP knowledge, education, and experience in palliative care emergencies |  |  | PCP knowledge, education, and experience in palliative care emergencies is an enabler to the delivery of palliative care by PCPs |  |
| Chatfield et al. (2017) | hand hygiene knowledge | Hand hygiene compliance rates | Skill-gap | Healthcare workers report that hand hygiene knowledge was not necessarily lacking, nor necessarily a contributing factor in lower than ideal compliance rates | No relation |
| Cormican et al. (2023) | Competence (skills and knowledge) | Implementation of clinical practice guidelines for stroke rehabilitation | Skill-gap | Knowledge and skills barriers identified ranged from a lack of familiarity with theoretical knowledge behind guideline recommendation to a lack of knowledge and skills on how to modify and transfer guidelines to own local context and implement guideline recommendations. Reversely, greater knowledge of guideline content, clinical skills and experience using recommendations facilitates guideline implementation in practice | Positive relation, i.e., if competence is present implementation of guidelines are higher. In other words, if lack of competence is high, implementation of skill gap will be low, hence why this relation is reversely coded (i.e., ‘red’) in the overview of evidence table. |
| Craig et al. (2016) | Lack of knowledge | Six key clinical behaviours in acute stroke care | Skill-gap | Barrier and enabler data regarding knowledge were available for two key clinical behaviours in acute stroke care: triage and thrombolysis. Both studies investigating triage reported that “stroke was not being recognised as a priority”. All three studies investigating thrombolysis identified barriers associated with lack of knowledge including guideline unfamiliarity, awareness, or failure to react to deviations in guidelines. Another study related more to procedural knowledge such as uncertainty with respect to the patient selection criteria for thrombolysis. | Negative relation |
|  | Lack of skills | Six key clinical behaviours in acute stroke care | Skill-gap | Lack of skills was reported in relation to the triage, thrombolysis and swallow care behaviours (as key clinical behaviours), but not for the transfer behaviours. |  |
| De Angelis et al. (2016) | Lack of knowledge | Spontaneous reporting of adverse drug reactions | Skill-gap | Knowledge is one of the intrinsic factors that conditions nurses’ reporting. Results show a lack of knowledge of pharmacovigilance, pharmacology, what constitutes an ADR, how to define an adverse drug reaction, or how to report them | Negative relation |
| Egerton et al. (2017) | Lack of knowledge/ feeling under-prepared | Management of osteoarthritis in line with clinical practice guidelines | Skill-gap | Clinicians lack knowledge about recommended practice, such as not being familiar with specific treatments recommended in clinical practice guidelines. Moreover, the data suggests that some clinicians perceive they are inadequately prepared in terms of knowledge to treat patients with osteoarthritis | Negative relation |
| Fekonja et al. (2023) | Skills | Patient safety during triage in the emergency department  *(categorized under ‘aggregated/unspecified patient safety’)* | Skill-gap | Personal capabilities, such as skills, influences the potential safety of patients, for example as these allow triage nurses to adapt to difficult emergency situations. Triage requires from the nurse (…) critical thinking, and interprofessional communication skills (…). | Positive relation. Reversely coded (i.e. ‘red’) in the overview of evidence table, illustrating a lack of skills or knowledge, rather than a presence of skills or knowledge |
|  | Knowledge and clinical capability |  | Skill-gap | Knowledge and clinical capability are crucial for patient safety in the triage process. Differences in the level of knowledge are sometimes reflected in the decisions of triage nurses. The skill level of triage nurses directly affects the triage process, namely the accuracy of patient assessment, which means that it can provide patients with adequate quality triage care or otherwise jeopardise their safety. Knowledge and skills in the triage process mostly refer to knowledge of triaging the patient, sufficient clinical knowledge in the field of the physiopathology of diseases and emergencies, clinical skills to perform correct and rapid clinical measures, the ability to check patient history and perform physical examinations. |  |
| Gallione et al. (2022) | Knowledge and skills | Implementation of Clinical Practice Guidelines (CPGs) | Skill-gap | Knowledge and skills may represent a facilitating factor for the implementation of CPGs. On the other hand, their lack or inaccessibility could represent an important barrier (aggregate result of 34 studies) | Positive relation. Reversely coded (i.e. ‘red’) in the overview of evidence table, illustrating a lack of skills or knowledge, rather than a presence of skills or knowledge |
| Hamed & Konstantinidis (2022) | Lack of skills or knowledge | Incident reporting | Skill-gap | Most of the included studies evidenced a lack of skills, knowledge, and training required to take part in effective incident reporting | Negative relation |
| Houghton et al. (2020) | Poor knowledge of risk factors associated with specific infections (SARS, TB) | Adherence to Infection Prevention and Control (IPC) guidelines for respiratory infectious diseases | Skill-gap | Poor knowledge of risk factors associated with specific infections (SARS, TB) impacted on the healthcare workers’ understanding of policies and subsequently their adherence to IPC guidance. | Negative relation |
| Jun et al. (2016) | Knowledge | Use of clinical practice guidelines | Skill-gap | Nurses had general knowledge of clinical practice guidelines but lacked familiarity or awareness of specific clinical practice guidelines to be able to incorporate them into practices (barrier). Knowledge was also found to be a facilitator for the use of CPGS. | Positive relation. Reversely coded (i.e. ‘red’) in the overview of evidence table, illustrating a lack of skills or knowledge, rather than a presence of skills or knowledge |
| Kelly et al. (2023) | Knowledge of standards | Implementation of (inter)nationally endorsed health and social care standards | Skill-gap | Knowledge of standards is identified as a high confidence enabler for implementation of (inter)nationally endorsed health and social care standards. Reversely, a lack of knowledge, awareness or understanding of the standards was the most frequently reported barrier to implementation of standards. Knowledge gaps related to the rationale for standards, their content, expectations and knowledge of available support tools. | Positive relation. Reversely coded (i.e. ‘red’) in the overview of evidence table, illustrating a lack of skills or knowledge, rather than a presence of skills or knowledge |
| McFadden et al. (2020) | Lack of knowledge, skills and competence | Provision of high quality-midwifery care (quality in line with Quality Maternal and Newborn Care (QMNC) and WHO standards for improving quality of maternal and newborn care in health facilities (e.g. preventing complications, first-line management of complications, routine evidence-based care, appropriate physical environment with adequate water) | Skill-gap | Authors report that a lack of knowledge, skills and behaviour of skilled birth attendants was a barrier to the provision of high-quality midwifery care | Negative relation |
| Ng et al. (2021) | Knowledge and skills | Adoption of a biopsychosocial approach to musculoskeletal pain  *(categorized under ‘adherence to guidelines’)* | Skill-gap | Knowledge and skill are identified as enablers for the adoption of a biopsychosocial approach to musculoskeletal pain, but a lack of knowledge and skills as a barrier for its adoption. Examples of barriers include inability to identify and manage psychosocial factors, inability to apply the biopsychosocial model holistically, and to understand the concept of evidence-based practice sufficiently. Moreover, nontechnical skills are identified as barriers, such as lack of communication, interpersonal and counselling skills. Examples of enablers include: the ability to build trust and rapport, be emphatic and validate the patients’ perspective, knowing how to manage conflicts and treatment goals, or the ability to use patient-centred communication, lay medical vocabulary and language to engage, motivate and provide explanations to patients. | Positive relation. Reversely coded (i.e. ‘red’) in the overview of evidence table, illustrating a lack of skills or knowledge, rather than a presence of skills or knowledge |
| Niño de Guzmán et al. (2020) | Lack of knowledge | Health care providers’ adherence to breast cancer clinical guidelines | Skill-gap | Factors that potentially impact on health care providers’ adherence to breast cancer clinical guidelines include a lack of knowledge, such as finding it difficult to interpret recommendation | Negative relation |
| Okuyama et al. (2014) | Perceived lack of sufficient knowledge | Speaking-up behaviour | Skill-gap | Perception of a lack of sufficient knowledge is a barrier to speaking up, as health care professionals tend to hesitate to speak up when they feel they are not adequately informed. | Negative relation |
|  | Communication skills | Speaking-up behaviour | Skill-gap | Health care professionals’ communication skills, such as the ability to use assertive and critical language, have an influence on self-confidence and speaking-up behaviour. | Positive relation. Reversely coded (i.e. ‘red’) in the overview of evidence table, illustrating a lack of skills or knowledge, rather than a presence of skills or knowledge |
| O’rorke et al. (2022) | Clear communication | Patient satisfaction | Skill-gap | Patient satisfaction was lower when patients were unclear on the roles of each member of their care team, underscoring the need of the surgeon to introduce each team member. Patient satisfaction was also greater when surgeons were able to set expectations by clearly describing individual care plans and the risks and potential short- and long-term outcomes of the procedure. Satisfaction was lower when surgeons who exhibited poor communication with other team members. One study did not find a significant difference in patient satisfaction when vascular surgeons showed patients their angiography pre-operatively versus when they did not incorporate imaging as a communication aid. | Positive relation. Reversely coded (i.e. ‘red’) in the overview of evidence table, illustrating a lack of skills or knowledge, rather than a presence of skills or knowledge |
|  | Communication that involves the patient | Patient satisfaction | Skill-gap | Encouragement of patient questions throughout the discussion improves patient satisfaction. Surgeons who adequately answered patient questions also experienced higher patient satisfaction ratings. Patients were also more satisfied with their treatment option when they perceived a greater level of autonomy-supporting communication (e.g. listening to how a patient would like to approach treatment before offering own opinion). Another study found that more decisional control, even if patients expressed less interest in having control over their treatment plan, correlated with a higher satisfaction with care. Patient satisfaction only increased when patients actively participated in their treatment decisions, in contrast to surgeons simply soliciting participation from their patients. |  |
|  | Demeaner and interpersonal skills | Patient satisfaction | Skill-gap | One study showed that patient satisfaction increased when surgeons shook the patients’ hand, introduced him/herself using his/her last name, and addressed the patient on a first name basis. Moreover, patients had a more satisfying experience when surgeons were personable and developed rapport (e.g. by expressing in interest in learning about the patients through asking about comorbid illnesses, occupation, family and home life). |  |
| Parajuli & Hupcey (2021) | Providers’ lack of knowledge about palliative care in oncology | Palliative care referral (in line with recommendations/guidelines) for persons with cancer | Skill-gap | Providers’ lack of knowledge about Palliative care is perceived as a barrier to PC referral for persons with cancer. For example, providers equated PC with hospice, believed that PC could not be concurrent with cancer therapy, should be provided to patients with life expectancy of less than 3 months and described PC services only as pain management services. Providers also lacked information about the components of PC, lacked knowledge on opportunities and delivery of PC or EOL care, locally available services or local outpatient PC services. | Negative relation |
|  | Insufficient/poor communication skills | Palliative care referral (in line with recommendations/guidelines) for persons with cancer | Skill-gap | Insufficient/poor communication skills were also identified as a barrier to PC referral. |  |
| Parry et al. (2015) | Clinical expertise (measured as knowledge of medications or ability to anticipate potentially adverse situations | Medication administration error | Skill-gap | One article found that individual RNs with greater knowledge were less likely to violate medication protocols whilst two other studies identified knowledge, such as dose calculation knowledge, contributed to medication administration error. Yet another article found that expertise was significantly related to fewer non-severe errors. | Mixed evidence |
| Peng et al. (2023) | Insufficient knowledge of missed nursing care | Reporting missed nursing care | Skill-gap | Insufficient knowledge of missed nursing care is the main obstacle to nurses’ reluctance to report missed nursing care | Negative relation |
| Pitzer et al. (2024) | Insufficient knowledge about palliative care | Access to palliative care | Skill-gap | 27 studies addressed insufficient knowledge by health professionals, mostly about palliative care, as a barrier to patients’ access to palliative care. | Negative relation |
|  | Self-confidence among non-palliative care clinicians to have sufficient palliative care competence | Access to palliative care | Skill-gap | Both low (one study) and high self-confidence (six studies) among non-palliative care clinicians to have sufficient palliative care competence has been reported as a barrier to patients’ access to palliative care | Negative relation. Reversely coded (i.e. ‘green’) in the overview of evidence table, illustrating a lack of self-confidence rather than a presence of confidence |
| Putri et al. (2024) | Lack of knowledge | Underreporting of adverse drug reactions | Skill-gap | Lack of knowledge on what, when, and to whom to report adverse drug reactions was identified as one of the major six reasons for underreporting of adverse drug reactions | Positive relation. Reversely coded (i.e. ‘red’) in the overview of evidence table, illustrating a lack of skills or knowledge, rather than a presence of skills or knowledge |
| Salmasi et al. (2015) | Lack of knowledge | Medication error | Skill-gap | 2/15 studies that report contributing factors to medication errors identify a lack of knowledge as a contributing factor to medication errors | Positive relation |
| Schroers et al. (2020) | Lack of medication knowledge | Medication administration error | Skill-gap | Lack of medication knowledge was commonly cited as a nurse perceived cause of medication administration error. Examples include lack of knowledge of safe dosages of medications, proper dilutions, and drug interactions. | Positive relation |
|  | Inability to properly calculate medication doses and infusion rates | Medication administration error | Skill-gap | Inability to properly calculate medication doses and infusion rates were also perceived by nurses to contribute to MAEs |  |
|  | Inability to use technology | Medication administration error | Skill-gap | Inability to use technology were also perceived by nurses to contribute to MAEs |  |
| Slade et al. (2016) | Minimal knowledge of the existence of guidelines or their content, and how they were derived | Implementation of Low Back Pain Clinical Practice Guidelines | Skill-gap | Most clinicians reported minimal knowledge of guideline content and how they were derived. Some were unaware of their existence. | Negative relation |
|  | Beliefs about knowledge limitation | Implementation of Low Back Pain Clinical Practice Guidelines | Skill-gap | GPs lacked confidence in their ability to assess LBP and provide evidence-based care, whereas allied health clinicians (chiropractors, OTs, and PTs) appeared more assured. | Partial relation, depending on the profession. |
| Smiddy et al. (2015) | Knowledge | Compliance with hand hygiene guidelines | Skill-gap | Health care workers’ knowledge of the appropriate manner to perform hand hygiene was linked with education and consequent practice. In the studies reviewed, some health care workers did not have the correct understanding of the importance of hand hygiene. | Positive relation. Reversely coded (i.e. ‘red’) in the overview of evidence table, illustrating a lack of skills or knowledge, rather than a presence of skills or knowledge |
| Thomas et al. (2019) | Lack of knowledge | Medication error | Skill-gap | Review identified lack of knowledge (from staff) as a contributory factor leading to medication errors. | Positive relation |
| Toomey et al. (2021) | Lack of practitioners’ awareness knowledge of the eye care area | Eye care delivery (in line with evidence-based practice) | Skill-gap | A lack of practitioners’ awareness knowledge of the eye care area was reported as a barrier to eye care delivery by 19 studies, whilst having awareness and knowledge of the eye care area was a facilitator to eye care delivery in 15 studies. Examples include knowledge deficits for diagnostic criteria, or the impact and management of depression in low vision patients. Knowledge of the guidelines was a predictor of delivery evidence-based eye care for both glaucoma and diabetic eye care. | Negative relation |
|  | Skills proficiency deficits | Eye care delivery (in line with evidence-based practice) | Skill-gap | Skills proficiency deficits was a barrier with issues noted in clinical, communication and other skills (research, business) as evidenced in 16 studies, whilst 8 studies found that proficiency in those areas was a facilitator. Predominant skills deficiency was in clinical skills, such as difficulty in examination (paediatrics). |  |
| Vaismoradi et al. (2020) | Lack of knowledge | Disclosure and reporting of practice errors | Skill-gap | One of the main barriers to disclosure and reporting of practice errors was a lack of knowledge by nurses on the process of reporting. | Negative relation |
| Vrbnjak et al. (2016) | Lack of knowledge | Reporting medication error | Skill-gap | Especially a lack of knowledge of the process of error reporting, an unawareness of the medication error reporting system (i.e. not knowing how to report an error), and uncertainty about ME definitions, influenced nurses’ medication error reporting | Negative relation |
|  | Lack of skills in error management | Reporting medication error | Skill-gap | A lack of skills in error management was also found to influence nurses’ medication error reporting |  |
|  | Knowledge deficit | Reporting near misses | Skill-gap | A knowledge deficit on the definition of a near miss was most reported as a barrier to reporting near misses, whilst knowledge deficits about the reporting process and systems were less commonly reported |  |
| Woo & Avery (2021) | Deficiency in knowledge | Voluntary error reporting (VER) | Skill-gap | Four studies revealed how nurses’ attitude concerning the unintentional omission of error reporting was due to their lack of knowledge to perceive and recognize events that constituted errors. Moreover, nurses perceived that adequate knowledge and abilities were enabling factors of VER. | Negative relation |
| Zhang et al. (2024) | Physician empathy as a communicative competence | Patients’ functional status, here: anxiety  *(categorized under ‘patient outcomes’)* | Skill-gap | Physician empathy is related to better patient outcomes (here: less patient anxiety) when measured by a researcher who observes encounters, but not when measured as clinician-perceived physician empathy. | Partial relation, depends on how physician empathy is measured (through observation or perception) |
|  | Physician empathy as a communicative competence | Patient satisfaction | Skill-gap | One study found no relation between physician self-perceived empathy and patient satisfaction. Another study that measured empathy by counting the number of emphatic verbal responses by physicians by a researcher (labelled “accurate empathy”) found a positive impact of empathy on patient satisfaction. Moreover, this author also found that absence of physician empathy has a negative effect on patients’ consultation satisfaction. |  |
