# Supplementary material for: Healthcare workers’ sustainable employability in relation to quality of care: an umbrella review
Source: BMJ Open. 2025 Sep 8;15(9):e095126. doi: 10.1136/bmjopen-2024-095126 (PMC12421189; doi:10.1136/bmjopen-2024-095126)
Supplement: Supplementary file 5 [file bmjopen-2024-095126.reviewer_comments.pdf]

PEER REVIEW HISTORY

BMJ Open publishes all reviews undertaken for accepted manuscripts. Reviewers are asked to complete a checklist review form and are provided with free text boxes to elaborate on their assessment. These free text comments are reproduced below.

ARTICLE DETAILS

Title (Provisional)

Health care workers' sustainable employability in relation to quality of care: an umbrella review

Authors

van de Voort, Iris; Leistikow, Ian; Weenink, Jan-Willem

VERSION 1 - REVIEW

**Reviewer**	**1**
**Name**	**Taylor, Cath**
**Affiliation**	**University of Surrey, School of Health Sciences**
**Date**	**15-Nov-2024**
**COI**	**None**

Well done on conducting an important and novel umbrella review of this important topic.

This is a very high quality well written paper describing an umbrella review of the relationship between sustainable employability and quality of care. The review was conducted with rigour using robust methodology (JBI), and the presentation of findings is very good - with a summary at the end of each written section, and with Table 2 and Figure 2 providing a very useful overview of the relationships between variables and consistency (or not) between studies, including providing clear visuals about where research attention has been focused (and the gaps).

My comments are very minor:

1. In the 'strengths and limitations' bullets the second bullet says moderate, high and very high quality reviews were included, but in the abstract it says only high quality reviews were included.

2. The first paragraph in the background is a little difficult to follow. I would suggest that it may read better if the second and third sentences, instead of being positioned as presenting contrasting views (on the one hand etc) that instead these points are presenting the 'context' that is referred to in the previous sentence. That the given employment context is

one of high and increasing demand for services against a backdrop of high vacancies/short staff and inadequate resources?

3. Methods - very robust and strong section, but would be improved by a clear statement of objectives/review question.

4. Line 24, page 8 - should this also say 'relevant SE outcomes' as well as relevant QoC outcomes (when stating the data that were extracted)?

5. The review included a number of qualitative reviews, which I presume were more focussed on how/why concepts were related rather than 'if' they were. Perhaps more of this could be drawn upon?

**Reviewer**	**2**
**Name**	**Shringarpure, Kalpita**
**Affiliation**	**Medical College Baroda, Department of PSM**
**Date**	**15-Jun-2025**
**COI**	**None**

Dear Authors

This is an extremely important topic in terms of healthworker satisfaction and its effects.

However,

1. The methodology needs detailing

2. Results can be made more concise along with a figure

3. Discussion highlights only burnout. Other aspects need to be discussed as well.

Other comments have been added in the attached file

Regards

**** **The reviewer provided a marked copy with additional comments. Please contact the publisher for full details.** ****

VERSION 1 - AUTHOR RESPONSE

**Reviewer 1**	
**1.** In the 'strengths and limitations' bullets the second bullet says moderate, high and very high quality reviews were included, but in the abstract it says only high quality reviews were included.	We understand the confusion. To avoid confusion, we have omitted the labels ‘moderate, high and very high quality’, and simply explained in the results (quality appraisal section) how many

	reviews (in %) scored positive on how many items. In addition, we also added a sentence in the method section that the JBI manual does not specify when a review is considered high quality, and that as a result, we pre-established our own criteria for concluding so.
**2.** The first paragraph in the background is a little difficult to follow. I would suggest that it may read better if the second and third sentences, instead of being positioned as presenting contrasting views (on the one hand etc) that instead these points are presenting the 'context' that is referred to in the previous sentence. That the given employment context is one of high and increasing demand for services against a backdrop of high vacancies/short staff and inadequate resources?	Excellent suggestion, we have edited accordingly.
**3.** Methods - very robust and strong section, but would be improved by a clear statement of objectives/review question.	We have included both an aim and a research question in the first sentence of the method section.
4. Line 24, page 8 - should this also say 'relevant SE outcomes' as well as relevant QoC outcomes (when stating the data that were extracted)?	With relevant SE variables, we mean HCWs' variables that fit within the theoretical operationalization of SE that we use (so being an exact indicator of SE or considered a synonym). To clarify this and match it with the results section, we have rephrased 'relevant SE variables' to: 'SE indicators'.
5. The review included a number of qualitative reviews, which I presume were more focussed on how/why concepts were related rather than 'if' they were. Perhaps more of this could be drawn upon?	Although it would be interesting to understand more about how and why concepts were related, we found that in practice, qualitative reviews mostly explored barriers or facilitators in relation to a specific QoC outcome (e.g. career dissatisfaction as a mentioned reason for missed nursing care). As the reviews that we included already concerned aggregated data from primary studies, reasons as to how or why were hardly explored or mentioned in these reviews. We do not consider that a problem for the aim of this review, which is to present an overview of the ways SE indicators are related to QoC outcomes, whether established through quantitative analysis or qualitative research. We do think that

	future studies could focus more on how and why concepts are related and make this recommendation in the discussion.
**Reviewer 2**	
**1.** The methodology needs detailing	We have commented on your in-text remarks regarding the methodology below.
2. Results can be made more concise along with a figure	We have included a figure (and an overview table) of results as separate files.
3. Discussion highlights only burnout. Other aspects need to be discussed as well.	In the discussion, we specifically highlight burnout because we want to highlight the point of how a vast number of reviews on burnout (sometimes with the exact same aim or research question) may overshadow other important manifestations of HCW impairment, such as moral injury, equally important for HCWs' SE. Furthermore, we had to choose among all of our findings which findings in particular stood out (which were not necessarily as expected), and required further nuance and context. We chose the relation between burnout & patient satisfaction, but also the inconsistent relationship between surgeon fatigue (another SE indicator) and patient outcomes.
4. Abstract: comment: "full form" (referring to the JBI Critical Appraissal Form)	We have written JBI in full in the Abstract: "Joanna Briggs Institute"
5. Abstract: comment: "can include the effect size values here".	We did not conduct a meta-analysis for overall or average effect sizes for the 50 relations that we reported upon. If individual reviews reported effect sizes (mind that not all reviews did so, but most meta-analyses did) for a relation between an SE indicator and a QoC outcome then these can be found in supplemental file 4. However, as some relations were reported upon in multiple different reviews, effect sizes may differ between reviews.
6. Abstract: comment: "Conclusion to be added"	We have restructured the abstract in accordance with the instruction of the editor (see above) and changed the heading 'discussion' to 'conclusions'
7. Method: comment: "How many review articles were obtained?"	We have added that information in the first paragraph of the results section:

	"The literature search yielded 7017 unique records after duplicates were removed, of which 78 articles met inclusion criteria after screening title/abstract and full text articles. In addition, the backwards and forwards citation search retrieved another 38 records. In total 116 articles met the inclusion criteria and were subjected to quality appraisal (see Figure 1 for a depiction of the process and exclusion reasons based on the PRISMA flowchart). Of 116 articles, 61 articles did not meet our pre-determined quality criteria, meaning the final inclusion of this review amounts to 55 articles."
8. Method: comment: "What were the degrees and qualifications of the authors? Were they trained in systematic reviews? Duration of screening?"	The first author is a phd candidate and has a background in organizational learning & development, and strategic occupational health. The second author is trained as a physician, but currently works as a professor in health care regulation. The third author is an associate professor in quality, safety and professional work in health care. Both the second and third author have extensive experience conducting (and publishing) systematic and other literature reviews. The first author was supervised by the second and third author whilst conducting the review and did follow a course on conducting literature reviews prior to conducting this review. We have added information about the background of the research team in the methods section (design).<br><br>**Duration of screening:** Our literature search covered literature up until 10 of May, 2024. After that we screened title/abstract and full-text. This phase was concluded at the beginning of July. Thereafter, we conducted quality appraisal and data extraction. We have added this information in the manuscript.
9. Method: comment: "flowchart for both; screening and quality assessment to be included along with reasons for exclusion. Was any software used for screening?"	A prisma flowchart can be found as a separate attachment (figure 1) and includes a separate flowchart for the systematic search using our search string (left column) and a separate flowchart for

	our backwards and forwards search (right column). The flowchart lists exclusion reasons in the search and quality appraisal stage. EndNote was used for screening. We have added in the methods section that we used EndNote.
10. Results: comment: “mention the data extraction period? Study period?”	We conducted literature searches up until May, 10th 2024 and have added this date in the manuscript to the method section (under heading ‘Data sources and searches). In addition, data extraction after the search and quality appraisal was conducted in July and Augustus of 2024
11. Results: comment: “usually umbrella reviews include only systematic reviews. Any reason why other reviews were included? How were meta [comment in text is cut short]”	Traditionally, umbrella reviews focus on systematic reviews and meta-analyses, but the Joanna Briggs Institute also provides guidance on including qualitative synthesis. We included both quantitative and qualitative reviews as we aimed for a comprehensive overview of available evidence (regardless of the type of evidence) on the relationship between sustainable employability of health care workers and quality of care outcomes. In this way, researchers and practitioners get the full picture and can see what gaps in the literature remain.
12. Results: comment: “how was outcomes in terms of patient related to providers? Details to be provided in introduction and discussion”.	The relations between patient outcomes and providers explored was always a direct one. For example, the relation between burnout (of providers) and (unfavourable) patient outcomes. We have added the word ‘direct’ relationship in both the introduction and discussion to underline this.
**Additional changes by authors in revised manuscript**	
**Supplemental file 4**	We noticed that supplemental file 4 had no title on the page itself, which might make it hard for the reader to locate the file. We have added this. Also, in the manuscript we referred to ‘supplementary file 4’ (wrongly spelled). We have changed this into ‘supplemental file 4’.

VERSION 2 - REVIEW

**Reviewer**	**2**
**Name**	**Shringarpure, Kalpita**
**Affiliation**	**Medical College Baroda, Department of PSM**
**Date**	**06-Jul-2025**
**COI**	

The authors have addressed the reviewer comments appropriately.
